# Association between microbiological risk factors and neurodegenerative disorders: An umbrella review of systematic reviews and meta-analyses

**DOI:** 10.3389/fpsyt.2022.991085

**Published:** 2022-09-23

**Authors:** Xin Wang, Deming Jiang, Tianxiong Li, Xiao Zhang, Ran Wang, Song Gao, Fengyi Yang, Yan Wang, Qi Tian, Chunrong Xie, Jinghong Liang

**Affiliations:** ^1^Department of Neurology, Beijing Huairou Hospital of Traditional Chinese Medicine, Beijing, China; ^2^Department of Neurology, Xuanwu Hospital, Capital Medical University, Beijing, China; ^3^Surgery Centre of Diabetes Mellitus, Peking University Ninth School of Clinical Medicine (Beijing Shijitan Hospital, Capital Medical University), Beijing, China; ^4^Department of Maternal and Child Health, School of Public Health, Sun Yat-sen University, Guangzhou, China

**Keywords:** microorganism, umbrella review, neurodegenerative disorders, meta analyses, observational studies

## Abstract

The role of microbiological factors in the development of neurodegenerative diseases is attracting increasing attention, while the relationship remains debated. This study aimed to comprehensively summarize and evaluate the associations between microbiological factors and the risk of neurodegenerative disorders with an umbrella review. PubMed, Embase, and the Cochrane library were used to search for papers from the earliest to March 2021 for identifying meta-analyses and systematic reviews that examined associations between microbiological factors and neurodegenerative diseases. AMSTAR2 tool was employed to evaluate the methodical quality of systematic reviews and meta-analyses. The effect size and 95% confidence interval (95% CI) were recalculated with a random effect model after the overlap was recognized by the corrected covered area (CCA) method. The heterogeneity of each meta-analysis was measured by the *I*^2^ statistic and 95% prediction interval (95% PI). Additionally, publication bias and the quality of evidence were evaluated for all 37 unique associations. Only 4 associations had above the medium level of evidence, and the rest associations presented a low level of evidence. Among them, helicobacter pylori (HP), infection, and bacteria are associated with Parkinson's disease (PD), and the other one verifies that periodontal disease is a risk factor for all types of dementia. Following the evidence of our study, eradication of HP and aggressive treatment of periodontitis are beneficial for the prevention of PD and dementia, respectively. This umbrella review provides comprehensive quality-grade evidence on the relationship between microbial factors and neurodegenerative disease. Regardless of much evidence linking microbial factors to neurodegenerative diseases, these associations are not necessarily causal, and the evidence level is generally low. Thus, more effective studies are required.

**Systematic review registration:**

https://www.crd.york.ac.uk/PROSPERO/#searchadvanced, PROSPERO, identifier: CRD42021239512.

## Introduction

With the aging process, neurodegenerative diseases, such as dementia, PD, motor neuron disease, and multiple system atrophy, are increasingly challenging to global public health. In 2016, the global number of individuals who lived with dementia was 43.8 million, increasing from 20.2 million in 1990. This increase of 117% contrasted with a minor increase in age-standardized prevalence of 1.7%, from 701 cases per 100,000 population in 1990 to 712 cases per 100,000 population in 2016 (1). Furthermore, the fastest growing neurological disorder in the world is PD. From 1990 to 2015, the number of people with PD doubled to over 6 million. This number is projected to double again to over 12 million by 2040 (2). Therefore, it is of great significance to prevent the occurrence or delay the progression of neurodegenerative diseases at an early stage in healthcare systems worldwide under a shortage of therapeutic drugs.

More and more risk factors are being investigated, such as age, genetics, environment, diet, obesity, and drugs. Increasing attention has been paid to the microorganism, which is an independent risk factor. For example, Sun et al. reported that fecal microbiota transplantation alleviated microbial dysbiosis and finally exerted neuroprotective effects on the methyl- 4-phenyl-1,2,3,6-tetrahydropyridine (MPTP) mouse model (3). FLZ is a novel squamous amide derivative effective in many PD models. Notably, FLZ inhibits systemic inflammation by reducing intestinal inflammation and intestinal barrier damage, and finally achieves a protective effect on the rotenone-induced PD model in mice (4). These results suggest that PD may be caused by intestinal pathogenic factors (5), especially intestinal flora imbalance. In addition, Judith Miklossy even believed that the senile plaques in Alzheimer's disease (AD) are composed of spirochetes, and spirochetes produce biofilms. She has applied various methods such as histochemistry, immunohistochemistry, *in situ* hybridization techniques, and TUNEL tests to confirm the biofilm nature of the senile plaques (6, 7). Another animal study in Japan demonstrated that oral administration of *Pseudomonas gingivalis* for 5 months in adult WT (Wild type) mice induced AD-like pathology, including amyloidosis and neurodegeneration in hippocampal and cortical regions (8). However, the current studies on the mechanism of infection of neurodegenerative diseases are only limited to animal models, and it remains unknown whether the process of animal studies can be repeated in the human pathological process (9, 10). Furthermore, the existing studies on humans are observational, and there have been many meta-analyses and systematic reviews of the relationship between microbial factors and neurodegenerative diseases with the increase in the number of basic original studies. Some of these meta-analyses and systematic reviews even drew opposite conclusions owing to differences in the scope of the included studies and research methods. For example, some articles suggested that AD was not associated with herpes simplex virus type 1 (HSV-1) infection (11), while others demonstrated that AD was correlated with HSV-1 infection (12, 13). Additionally, most meta-analyses summarize one type of neurodegenerative diseases, such as PD and AD, rather than the whole neurodegenerative disease. Moreover, the microbial species studied in each meta-analysis are also limited. Hence, a comprehensive review of these meta-analyses and systematic reviews is imperative. An umbrella review allows comparison and contrast of the review results related to review questions. The most distinctive feature of an umbrella review is that this type of evidence synthesis only considers the highest level of evidence and thus provides decision-makers with the highest quality of available evidence relevant to the questions raised (14).

In this study, an umbrella review of existing systematic reviews and meta-analysis of evidence on microbiological risk factors for neurodegenerative diseases is performed to provide decision-makers with comprehensive, high-quality evidence on biological risk factors for neurodegenerative diseases, such as viruses, bacteria, and parasites.

## Methods

The umbrella review followed the guidelines for Preferred Reporting Items for Systematic reviews and Meta-Analyses (PRISMA) (15). The PRISMA checklist was presented in Appendix 1 in Supplementary material. The protocol of this umbrella review was registered in PROSPERO (the International Prospective Register of Systematic Reviews; ID: CRD42021239512).

### Eligibility criteria

Meta-analyses or systematic reviews satisfying the following criteria were selected: (1) systematic reviews or meta-analyses investigated the association of microbiological or infection factors and neurodegenerative disorders (cognitive decline, cognitive impairment, mild cognitive impairment (MCI), AD, all-cause dementia, PD, motor neuron disease, or multiple system atrophy); (2) studies were conducted on available Relative Risks (RR), Odds Ratio (OR), Hazard Ratio (HR), Standardized mean difference (SMD)/mean difference (MD); (3) articles written in English or Chinese were published in peer-reviewed scientific journals. PECO definitions: (1) The population included human participants aged 18 years and older. (2) Exposures were identified through a scoping search. The scoping search involved search terms for microbiological risk factors of neurodegenerative disease. Studies on the following microbiological risk factors were identified: infection, organism category (such as virus, chlamydia, spirochete, fungus, and intestinal flora). (3) The comparison group was composed of individuals who had not been exposed to microbiological risk factors in cohort studies or longitudinal randomized controlled trials, or who had not developed the neurodegenerative disease in case-control studies. (4) Outcomes of interest were neurodegenerative disease: AD, PD, cognitive decline, MCI, or dementia.

Studies were excluded based on the following exclusion criteria: (1) no quantitative synthesis of the single study results was performed; (2) studies with duplicate publications on the same exposure and outcome; (3) guidelines, narrative reviews, literature reviews, genetic studies, and animal studies.

### Search strategy

Two researchers (XW, DMJ) independently searched systematic reviews and meta-analyses in three electronic databases (Medline, EMBASE, and Cochrane Library) using a search strategy according to predefined inclusion and exclusion criteria. All language publications were searched without any restriction of countries from the earliest date to March 2021. The search terms consisted of terms of exposure such as “Organisms Category,” “Infections,” “virus,” “intestinal flora,” “Gingivitis,” “Bacterial Infections,” “microorganism,” “H pylori,” “toxoplasma gondii,” “Herpes virus,” “EB virus,” “fungus,” “spirochete,” “*Porphyromonas gingivalis*,” “Gum infection,” “Chlamydia,” “Pneumonia,” outcomes such as “Neurodegenerative Diseases,” “dementia,” “Parkinson,” and “cognitive,” “Alzheimer Disease,” “Multiple System Atrophy,” “Lewy Body Disease,” “Motor Neuron Disease,” and terms referring to the study design including “systematic review” or “meta-analysis,” “Cochrane Database Syst Rev,” “pooling,” “clinical trial overview,” “Systematic Reviews as Topic,” “Meta-Analysis as Topic.” The full search strategy of PubMed is exhibited in Appendix 2 in Supplementary material. The search for other databases is similar. Additionally, disagreements between the 2 researchers were resolved by consensus with the third author (JHL). Reference lists of identified studies were screened for further relevant meta-analyses.

### Study selection and data extraction

Two researchers (XW, TXL) independently performed the study selection and data extraction from each included meta-analysis using a standardized form, which included the first author's last name, year of publication, type of studies included, number of databases, time frame for inclusion in the study, guidelines for reference, methods for quality assessment and publication bias of the original studies, heterogeneity, number of participants, country or region, number of original articles, and funds to support. Discrepancies were solved by consensus.

For every primary study included in every meta-analysis, the same two researchers (XW, TXL) independently extracted the data: name of the study, type of exposure(s) and comparisons, type of outcome(s), the number of cases and total participants, combined RR, or OR, or HR, or SMD/ MD and corresponding 95% CIs. No data were available in the meta-analysis, and the data were extracted from the original articles. Discrepancies were solved by consensus with the other two researchers (JHL, CRX).

### Quality assessment

AMSTAR2 is an instrument used in assessing the methodological quality of systematic reviews, involving 16 items (16). There are seven critical items: (1): Did the report of the review contain an explicit statement that the review methods were established prior to the conduct of the review and did the report justify any significant deviations from the protocol? (2): Did the review authors use a comprehensive literature search strategy? (3): Did the review authors provide a list of excluded studies and justify the exclusions? (4): Did the review authors use a satisfactory technique for assessing the risk of bias (RoB) in individual studies that were included in the review? (5): If meta-analysis was performed, did the review authors use appropriate methods for the statistical combination of results? (6): Did the review authors account for RoB in primary studies when interpreting/discussing the results of the review? (7): If they performed quantitative synthesis did the review authors carry out an adequate investigation of publication bias (small study bias) and discuss its likely impact on the results of the review? (16). The quality of the included meta-analysis and systematic review was divided into four grades: high, moderate, low, and critically low. Two reviewers (XW, RW) rated the methodological quality of the systematic reviews with the AMSTAR2 quality appraisal instrument. In the case of disagreements and failed consensus, a decision was reached by consulting a third reviewer (JHL).

### Overlapping reviews

Associations assessed in two or more reviews overlapped if the same exposure and outcome were evaluated (17). Merging results from reviews with overlapping associations could lead to the inclusion of primary studies more than once and thus overestimate the effect of the original research (18). Concerning overlapping associations in literature (that is, investigating the same exposure and outcome), a graphical cross-tabulation (citation matrix) of the overlapping systematic reviews (in columns) and the included primary studies (in rows) was generated (19). With a citation matrix, the degree of overlap can be quantified by the CCA method. CCA, expressed as a percentage, is calculated as (N–r)/(rc–r), where N denotes the number of publications included in evidence synthesis (or the number of ticked boxes in the citation matrix), *r* represents the number of rows, and c refers to the number of columns. Overlap is categorized as very high (CCA > 15%), high (CCA 11–15%), moderate (CCA 6–10%), or slight (CCA 0–5%). CCA is a validated method of quantifying the degree of overlap between two or more reviews and assists the decision process in handling overlaps.

All non-overlapping systematic reviews meeting the inclusion criteria were included in the analysis. Otherwise, the overlap between reviews was managed as follows.

If a high degree of overlap (CCA ≥ 11%) between two or more reviews was found, preference was given to the review that (in hierarchical order) had the highest rating and, was higher methodological quality levels with AMSTAR2 quality assessment tools; was most recent; supplied pooled effect estimates or conducted a meta-analysis; had the highest number of studies or participants. If a slight or moderate degree of overlap (CCA ≤ 10%) was observed, both reviews were retained, and the findings were compared.

### Statistical analysis

A standardized method was applied to the umbrella review. The original data of each forest map satisfying the criteria in the meta-analysis were extracted, and the original data were re-analyzed. The summary effect sizes, 95% CI, and *p* values were estimated using random effect models (Der Simonian Laird method). Besides, the 95% PI was also estimated. It accounted for the between-study heterogeneity and evaluated the uncertainty for the effect that would be expected in a new study addressing that same association (20). Between-study heterogeneity was quantified using the *I*^2^ metric. *I*^2^ values exceeding 50% indicated significant heterogeneity. The range of *I*^2^ quantifies the variability in effect estimates ascribed to heterogeneity rather than sampling error (21). Additionally, small-study effects (namely, whether smaller studies tend to give substantially larger estimates of effect size compared with larger studies) were assessed by Egger's regression asymmetry test (22). *P*-value ≥0.1 indicated no evidence of small-study effects.

The p-curve approach was adopted to examine whether there is the possibility of *p*-value tampering in a meta-analysis. P-hacking reflected the deliberate tampering of data until statistically significant results were found, that is, *P*-value < 0.05. The P-curve method assumes that if a real effect exists, *P*-value should follow a right-skewed distribution. In contrast, a left-skewed distribution indicated a high likelihood of p-hacking, while a non-right-skewed distribution suggested that the finding lacked evidentiary value.

### Evaluation of the quality of evidence

The following categories were adopted:

#### Highly convincing evidence

Highly convincing evidence required highly statistically significant summary associations (*p* < 10^−6^ by random effects); more than 1,000 cases; the largest study was statistically significant (*P* < 0.05); not large heterogeneity (*I*^2^ < 50%); 95% PI not including the null; no evidence of small-study effects (*P* > 0.1); with evidential value and no evidence of p-hacking.

#### Highly suggestive evidence

Highly suggestive evidence required highly statistically significant summary associations (*p* < 10^−6^ by random effects); more than 1,000 cases; the largest study was statistically significant (*P* < 0.05).

#### Suggestive evidence

Suggestive evidence required only *p* < 0.001 (*p* < 10^−3^) by random effects and more than 1,000 cases.

#### Weak evidence

The nominally significant associations had weak evidence (*p* < 0.05 by random effects).

#### Not significant

No significance threshold was discovered for the random-effects meta-analyses (*P* > 0.05). Statistical analyses and evidence ratings were conducted in R, version 4.0.2.

## Results

### Literature search

**Figure 1:**
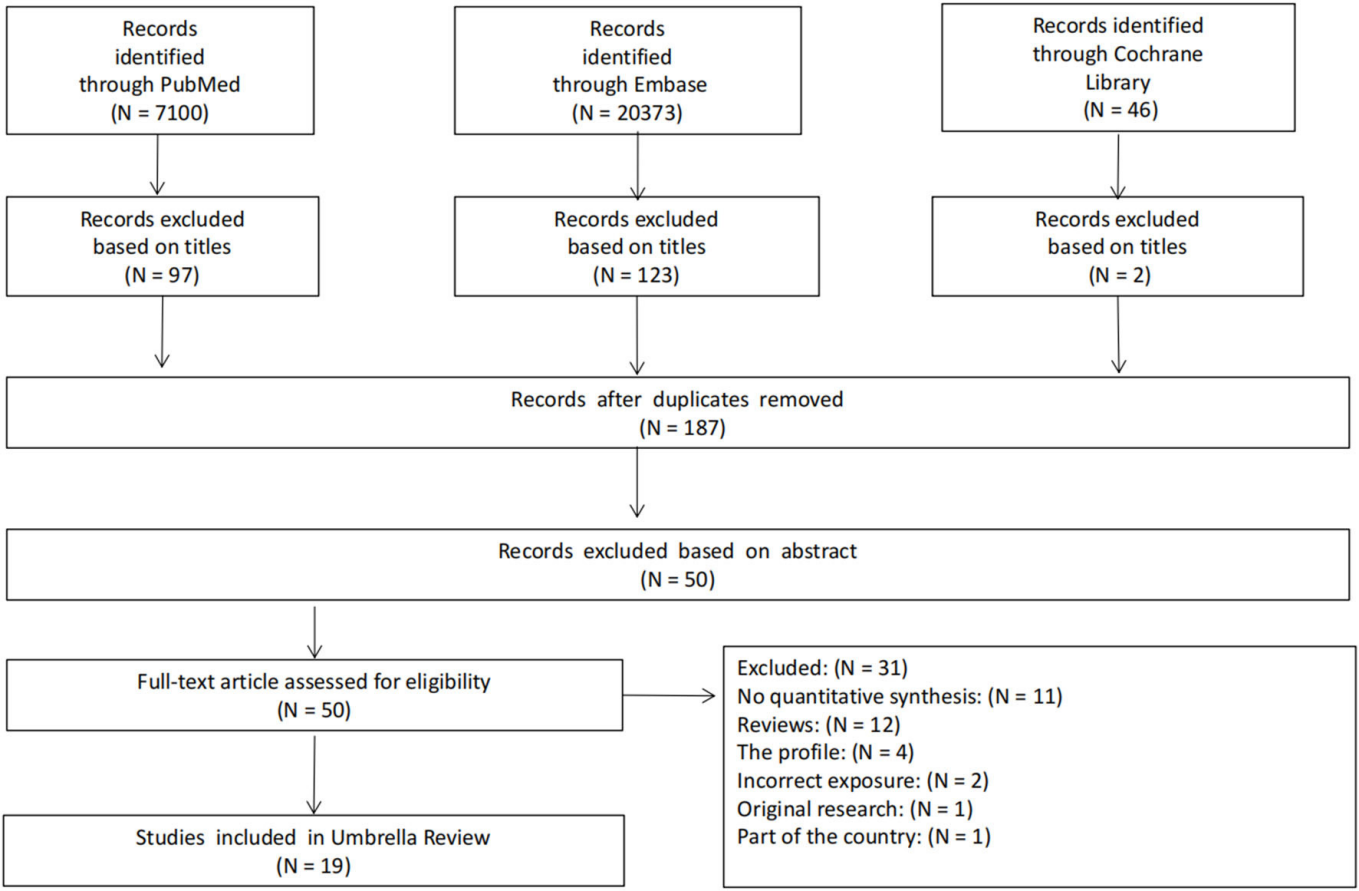
Flow diagram of literature search and study selection.

The search retrieved 27,519 articles. After the removal of duplicates and screening of titles and abstracts, 50 articles qualified for full-text screening. Nineteen meta-analyses for the umbrella review were identified following the inclusion and exclusion criteria. Figure 1 summarizes the study selection process. Appendix 3 in Supplementary material provides a list of studies excluded after the title and abstract screening (with reasons for exclusion).

The eligible articles were published between 2015 and 2020. The 19 articles corresponded to 62 unique meta-analyses: 30 on AD, 7 on all types of dementia, and 25 on PD. The overall characteristics of the 62 meta-analyses that were included in the umbrella review are summarized in Table 1. Thirty-seven unique risk factors were considered, 16 of which were studied in multiple articles. The median number of studies per meta-analysis was 5.5 (IQR, 2–36), and the median number of cases was 491 (IQR, 8–287773). Appendix 7 in Supplementary material provides a list of studies included.

**Table 1:** Overall characteristics of meta-analyses included in the umbrella review.

**References**	**Country**	**Exposure**	**Outcome**	**Comparison**	**Time frame**	**No. of** ** databases**	**Type of** ** study**	**No. of** ** included ** ** Studies**	**Effects** ** model**	**No. of participants/ no. of cases**	**MA** ** metric**	**Quality** ** appraisal** ** tool**	**Information** ** on** ** funding**	**Conflict** ** of** ** interests**
Pierantozzi et al. (23)	USA	HCV	PD	Without PD	From inception to May 2017	2	Case-control/cross-sectional/cohort studies	5	REM	7690987/66312	OR	NOS	No	No
Okoth et al. (18)	China	HP	PD	Without PD	January 1965 to October 2019	3	Case–control studies/cohort studies	9	REM	47601/1190	OR	NOS	No	No
		HCV	PD	Without PD				7	REM	>1998231/>28391	OR			
		Malassezia	PD	Without PD				2	FEM	>16354/>448	OR			
		*Chlamydophila pneumoniae*	PD	Without PD				2	FEM	>485/>213	OR			
		Measles	PD	Without PD				3	REM	3058/1235	OR			
		HBV	PD	Without PD				6	REM	>1375631/>19786	OR			
		Chicken pox	PD	Without PD				3	REM	2947/1124	OR			
		German Measles	PD	Without PD				2	FEM	1600/107	OR			
		HSV	PD	Without PD				4	REM	1883/393	OR			
		Infuenza	PD	Without PD				4	REM	21952/485	OR			
		Mumps	PD	Without PD				3	FEM	2643/820	OR			
		Scarlet fever	PD	Without PD				2	REM	>338/>8	OR			
		Whooping cough	PD	Without PD				2	REM	1429/414	OR			
Yang et al. (24)	China	Infection	PD	Without PD	From inception to December 2017	1	Case-control cohort studies	36	REM	7390674/287773	OR	NA	Yes	No
		Viruses	PD	Without PD				23	REM	6569826/133486	OR			
		Bacteria	PD	Without PD				9	REM	655977/135953	OR			
Aromataris et al. (14)	China	Herpesviridae family in case control studies	AD	Without AD	The first available year to March 2019	3	Longitudinal cohort/nested case control/case control	34	REM	2050/1083	OR	NOS	Yes	No
		*Chlamydophila pneumoniae*	AD	Without AD				11	REM	740/389	OR			
		Herpesviridae family in cohort and nested case control studies	AD	Without AD				9	REM	12166/1406	RR			
		HSV-1 in case control studies	AD	Without AD				18	FEM	1465/814	OR			
		HSV-1 in four prospective cohort and one nested case-control studies	AD	Without AD				5	FEM	11296/1020	RR			
		CMV in case control studies	AD	Without AD				6	REM	680/356	OR			
		CMV in cohort and nested case control studies	AD	Without AD				2	REM	1569/453	RR			
		HHV-6	AD	Without AD				4	FEM	456/204	OR			
		VZV	AD	Without AD				3	FEM	143/70	OR			
		EBV	AD	Without AD				2	FEM	297/112	OR			
		HP in case control studies	AD	Without AD				4	REM	973/610	OR			
		HP in cohort studies	AD	Without HP				3	FEM	94107/>1031	RR			
		Spirochetes	AD	Without AD				3	REM	89/52	OR			
Pieper et al. (17)	Australia	Spirochetes	AD	Without AD	MEDLINE (from 1950), PubMed (from 1946), EMBASE (from 1949) and Google Scholar (from 1993)	4	23 case-control studies/3 case series/1 randomized controlled trial	13	REM	1204/723	OR	NA	NA	NA
		Spirochetes (conservative)	AD	Without AD				9	REM	460/236	OR			
		*Chlamydophila pneumonia*	AD	Without AD				11	REM	508/282	OR			
Higgins et al. (21)	UK	Sepsis (exclude studies from Taiwan)	Dementia	Without sepsis	From inception to 18 March 2019	10	Longitudinal study/randomized controlled trial data/case control studies	3	REM	448428/33760	HR	GRADE	NA	NA
		Sepsis (remove studies with high risk of bias)	Dementia	Without sepsis			3	REM	503938/50624	HR			
Lagoo et al. (25)	Iran	Toxoplasmosis	AD	Without AD	From inception to November 25th, 2018	7	Case control/Cross sectional	8	REM	3239/360	OR	NOS	NA	NA
Letenneur et al. (26)	Iran	Toxoplasmosis (IgG antibodies)	PD	Without PD	to September 30, 2018	4	Case-control	8	REM	1068/478	OR	NOS	Yes	No
		Toxoplasmosis (IgM antibodies)	PD	Without PD				3	REM	550/210	OR			
		Toxoplasmosis	AD	Without AD				4	REM	614/301	OR			
Laurence et al. (27)	China	Toxoplasmosis (IgG antibodies)	PD	Without PD	Inception to October 2018	5	Case-controlled	8	FEM	1086/452	OR	NOS	Yes	No
		Toxoplasmosis (IgM antibodies)	PD	Without PD				3	FEM	600/221	OR			
Braak et al. (28)	UK	Periodontal disease (seven adjust studies)	Dementia	Without periodontitis	From the earliest date to 7th November 2018	6	Cohort and case-control studies	7	REM	226628/21065	RR	NOS	Yes	No
		Periodontal disease (seven ajust studies and four unajust studies)	Dementia	Without periodontitis			11	REM	227098/21298	RR			
Braak et al. (29)	Spain	Periodontal disease (All studies)	AD	Without AD	To January 2016 MEDLINE via PubMed (1946 to present), EMBASE (1974 to present) and Web of Science (1990 to present)	3	Cross-sectional/case-control/cohort study	3	FEM	822/204	RR	NOS	Yes	No
Alvarez-Arellano et al. (30)	Brasil	Periodontal disease	Dementia	Without dementia	January 1st 1997 to September 2st, 2017	3	Case-control/ cross-sectional/ longitudinal/ cohort studies	4	REM	644/302		NOS	No	No
Bjarnason et al. (31)	Australia	Herpesviridae	AD	Without AD	June 18, 2014 (first 20 pages)	4	Case control/ cohort/ trigeminal ganglion analysis	33	REM	2893/1330	OR	NA	No	NA
		HSV-1	AD	Without AD				18	REM	1631/780	OR			
		HHV-6	AD	Without AD				5	REM	419/195	OR			
		CMV	AD	Without AD				4	REM	283/145	OR			
		VZV	AD	Without AD				2	REM	114/53	OR			
		EBV	AD	Without AD				3	REM	354/121	OR			
Zhao et al. (4)	UK	HSV-1	AD	Without AD	From inception to December 2017	7	43 case-control/ 13 cohort/one RCT	16	REM	869/482	OR	Cochrane collaboration approach	Yes	NA
		HSV-1	Dementia	Without dementia			17	REM	922/497	OR			
Pisa et al. (32)	China	HSV-1	AD	Without AD	Between 1990 and February 2020	3	Case control/Cohort/ prospective studies	21	REM	3566/1338	OR	NOS	No	No
		HSV-1 (APOE e4-positive)	AD	Without AD				7	REM	456/319	OR			
		HSV-1 (APOE e4-negative)	AD	Without AD				7	REM	1517/803	OR			
Mahami-Oskouei et al. (33)	Greece	HP	PD	Without PD	1 November 1996 to 13 November 2017	1	Case-control/RCT/ cross-sectional/ cohort	10	FEM	28492/5043	OR	NA	Yes	No
Demmer et al. (34)	China	HP	PD	Without PD	January 1983 to January 2017 in PubMed	3	Case-control/ cross sectional/ 4prospective studies	8	FEM	33125/4934	OR	NA	Yes	No
Wu et al. (13)	China	Small intestinal bacterial overgrowth (SIBO)	PD	Without PD	Up to September 2018	1	Case-control/cohort	5	REM	607/292	OR	A quality scoring system modified from the original version of the Agency for Healthcare Research and Quality	Yes	No
		HP	PD	Without PD				9	REM	46918/5066	OR			
		HP	AD	Without AD				8	REM	89314/1502	OR			
Lövheim et al. (35)	Israel	HP	Dementia	Without dementia	To January 2015	4	Cohort/ Case control/ Cross-Sectional	7	REM	85715/18145	OR	NA	Yes	No

MA, meta-analysis; NA, not available; NOS, Newcastle-Ottawa Scale; OR, odds ratio; RR, risk ratio; HR, Hazard ratio; UK, The United Kingdom; USA; The United States of America; HCV, hepatitis C virus; PD, Parkinson's disease; REM, Random effect model; HP, Helicobacter pylori; FEM, Fixed effect model; HBV, hepatitis B virus; HSV, herpes virus; AD, Alzheimer's disease; HSV-1, herpes simplex virus type 1; CMV, cytomegalovirus; HHV-6, Human herpes virus type 6; VZV, varicella zoster virus; EBV, Epstein Barr virus; GRADE, Grading of Recommendations, Assessment, Development and Evaluation; APOE e4, Apolipoprotein e4; RCT, Randomized Controlled Trial.

### Methodological quality

None of the 19 meta-analyses were rated as high methodological quality, while 12 meta-analyses were rated as moderate, 5 meta-analyses were rated as low, and 2 meta-analyses were rated as critically low. Specific information on the methodological quality of the 19 meta-analyses evaluated using ASMTAR 2 is provided in Appendix 4 in Supplementary material.

### Overlapping and non-overlapping associations

Sixteen reviews reported overlapping associations, including 42 unique meta-analyses. Overlapping associations included: HCV and PD, *n* = 2; HP and PD, *n* = 4; HSV-1 and AD, *n* = 5; Herpesviridae family infection and AD, *n* = 3; *Chlamydia pneumoniae* and AD, *n* = 2; CMV and AD, *n* = 3; HHV-6 and AD, *n* = 2; VZV and AD, *n* = 2; EBV and AD, *n* = 2; HP and AD, *n* = 3; Spirochetes and AD, *n* = 3; Sepsis and dementia, *n* = 2; Toxoplasmosis and AD, *n* = 2; Toxoplasmosis (latent infection IgG antibodies) and PD, *n* = 2; Toxoplasmosis (acute infection IgM antibodies) and PD, *n* = 2; periodontal disease and dementia, *n* = 3. Appendix 6 in Supplementary material provides the general characteristics of the meta-analyses with overlapping associations, including the decision to retain or excluding an association from the analysis.

Appendix 5 in Supplementary material lists 16 citation matrixes used to assess the degree of overlaps.

### Summary findings

**Figure 2:**
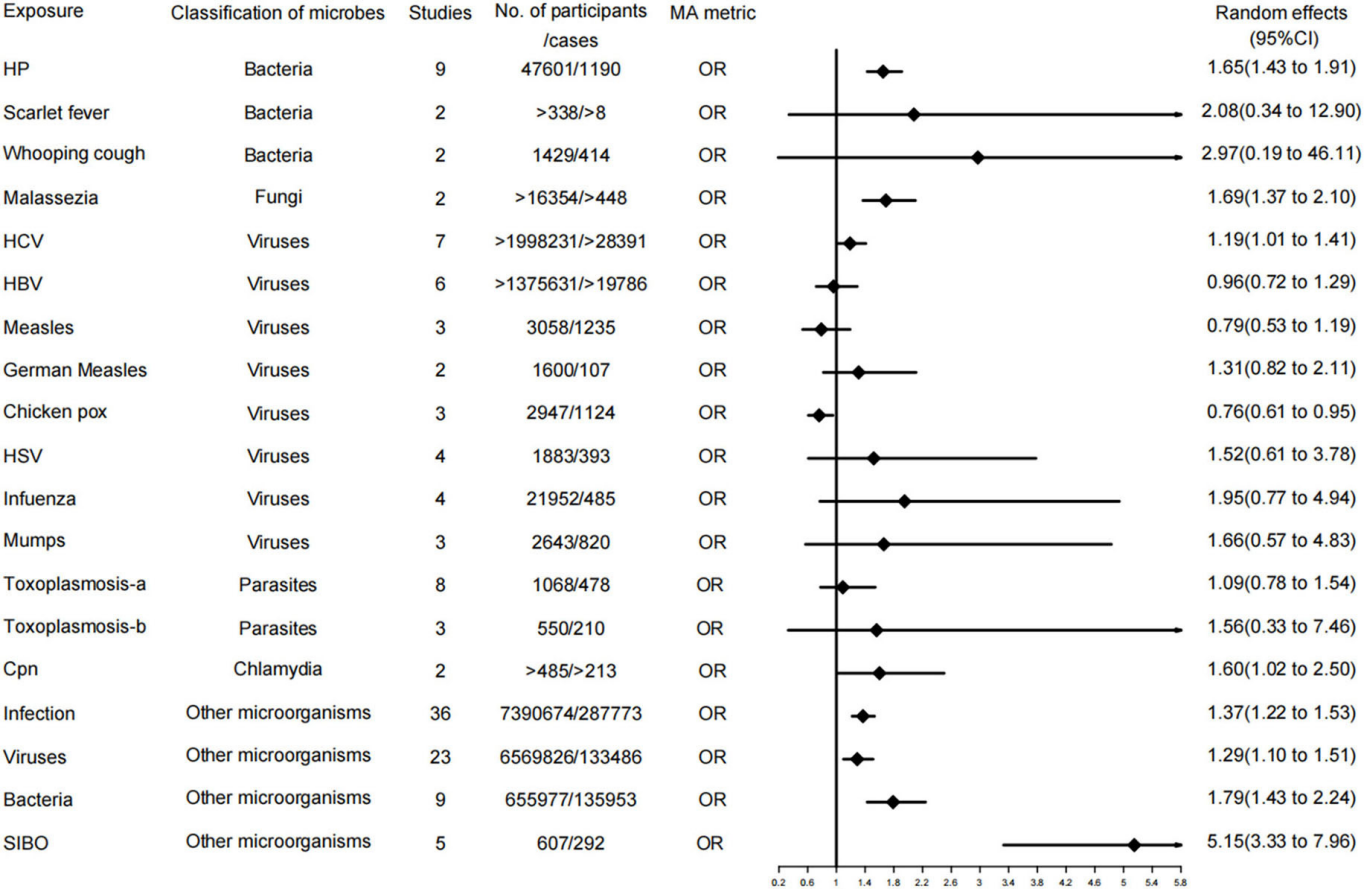
Forest plots and the level of evidence of the association of microbiological factors with Parkinson's disease. HP, *Helicobacter pylori*; HCV, hepatitis C virus; HBV, hepatitis B virus; HSV, herpes virus; Toxoplasmosis-a, Toxoplasmosis (lgG antibodies); Toxoplasmosis-b, Toxoplasmosis (lgM antibodies); SIBO, Small intestinal bacterial overgrowth.

**Figure 3:**
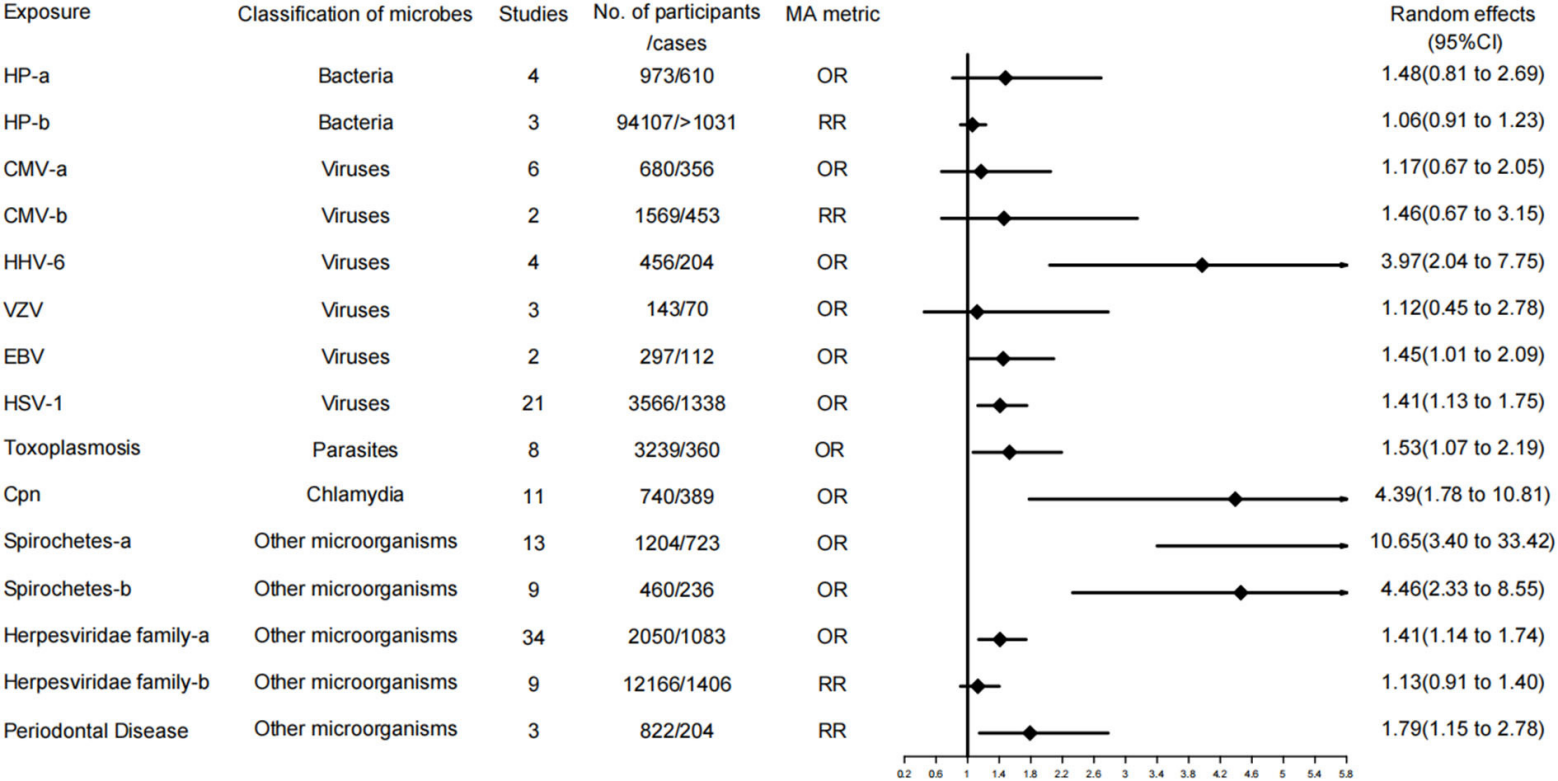
Forest plots and the level of evidence of the association of microbiological factors with Alzheimer's disease. Herpesviridae family-a, Herpesviridae family in case control studies; Herpesviridae family-b, Herpesviridae family in cohort and nested case control studies; CMV-a, CMV in case control studies; CMV-b, CMV in cohort and nested case control studies; HP-a, HP in case control studies; HP-b, HP in cohort studies; Spirochetes-a, all studies; Spirochetes-b, conservative studies; CMV, cytomegalovirus; HHV-6, Human herpes virus type 6; VZV, varicella zoster virus; EBV, Epstein Barr virus; HP, Helicobacter pylori; HSV-1, herpes simplex virus type 1; Cpn, Chlamydophila pneumoniae.

**Figure 4:**
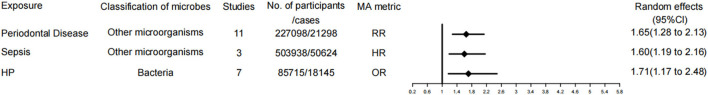
Forest plots and the level of evidence of the association of microbiological factors with dementia. HP, *Helicobacter pylori*.

A total of 37 independent meta-analyses on the relationship between microbial risk factors and neurodegenerative diseases were obtained after a literature search, methodological evaluation, and removal of overlap. Neurodegenerative diseases in the 37 independent associations were classified as PD, AD, and all types of dementia. In addition, the microbial risk factors for each disease were categorized into bacteria, fungi, viruses, parasites, chlamydia, and other microorganisms, and the risk factors that were not a single organism were classified as other microorganisms. Furthermore, the level of evidence was assessed for each of the 37 independent associations. Specific evidence levels and classification information are provided in Figures 2–4 and Table 2.

**Table 2:** Evidence of associations between microbial and neurodegenerative diseases.

**References**	**Classification of microbes**	**Exposure**	**Outcome**	**No. of primary studies**	**No. of participants/ no. of cases**	**OR/RR/HR**	**re-analysis 95% CI**	**P value**	**I^2^(%)**	**95% PI**	**Egger's test *p***	**Largest study significant**	**Evidential value for P-curve**	**Level of evidence**
Wang et al. (36)	Bacteria	HP	PD	9	47601/1190	OR	1.65 (1.43–1.91)	2.01E-11	0.5	1.38–1.98	0.0073	*P* < 0.05	Yes/no	II
Wang et al. (36)		Scarlet fever	PD	2	>338/>8	OR	2.08 (0.34–12.90)	0.4316	79	NA	NA	*P* > 0.05	NA	V
Wang et al. (36)		Whooping cough	PD	2	1429/414	OR	2.97 (0.19–46.11)	0.4368	85.1	NA	NA	*P* > 0.05	NA	V
Wang et al. (36)	Fungi	Malassezia	PD	2	>16354/>448	OR	1.69 (1.37–2.10)	1.50E-06	0	NA	NA	*P* < 0.05	NA	IV
Wang et al. (36)	Viruses	HCV	PD	7	>1998231/>28391	OR	1.19 (1.01–1.41)	0.0357	79.3	0.71–2.02	0.8542	*P* < 0.05	Yes/no	IV
Wang et al. (36)		HBV	PD	6	>1375631/>19786	OR	0.96 (0.72–1.29)	0.7835	90.5	0.35–2.66	0.675	*P* > 0.05	Yes/no	V
Wang et al. (36)		Measles	PD	3	3058/1235	OR	0.79 (0.53–1.19)	0.2632	60.2	0.01–63.47	0.9805	*P* > 0.05	NA	V
Wang et al. (36)		German Measles	PD	2	1600/107	OR	1.31 (0.82–2.11)	0.2627	0	NA	NA	*P* > 0.05	NA	V
Wang et al. (36)		Chicken pox	PD	3	2947/1124	OR	0.76 (0.61–0.95)	0.0137	0	0.18–3.14	0.0233	*P* > 0.05	NA	IV
Wang et al. (36)		HSV	PD	4	1883/393	OR	1.52 (0.61–3.78)	0.365	77.1	0.03–74.72	0.6168	*P* > 0.05	NA	V
Wang et al. (36)		Infuenza	PD	4	21952/485	OR	1.95 (0.77–4.94)	0.1575	93.1	0.02–163.18	0.3534	*P* > 0.05	NA	V
Wang et al. (36)		Mumps	PD	3	2643/820	OR	1.66 (0.57–4.83)	0.3506	94.6	0–1163047.02	0.1549	*P* > 0.05	NA	V
Bayani et al. (37)	Parasites	Toxoplasmosis-a	PD	8	1068/478	OR	1.09 (0.78–1.54)	0.6084	20.5	0.55–2.19	0.5874	*P* > 0.05	NA	V
Bayani et al. (37)		Toxoplasmosis-b	PD	3	550/210	OR	1.56 (0.33–7.46)	0.5765	0	0.00–39492.46	0.8105	*P* > 0.05	NA	V
Wang et al. (36)	Chlamydia	Cpn	PD	2	>485/>213	OR	1.60 (1.02–2.50)	0.0411	17.9	NA	NA	*P* > 0.05	NA	IV
Meng et al. (38)	Other microorganisms	Infection	PD	36	7390674/287773	OR	1.37 (1.22–1.53)	4.34E-08	83.8	0.80–2.33	0.3189	*P* < 0.05	Yes/no	II
Meng et al. (38)		Viruses	PD	23	6569826/133486	OR	1.29 (1.10–1.51)	0.0021	84.6	0.64–2.61	0.3071	*P* < 0.05	Yes/no	IV
Meng et al. (38)		Bacteria	PD	9	655977/135953	OR	1.79 (1.43–2.24)	3.93E-07	63.2	1.01–3.15	0.0023	*P* < 0.05	Yes/no	II
Fu et al. (39)		SIBO	PD	5	607/292	OR	5.15 (3.33–7.96)	1.62E-13	0	2.54–10.45	0.1522	*P* < 0.05	Yes/no	IV
Ou et al. (40)	Bacteria	HP-a	AD	4	973/610	OR	1.48 (0.81–2.69)	0.2024	72.3	0.12–18.72	0.0326	*P* > 0.05	NA	V
Ou et al. (40)		HP-b	AD	3	94107/>1031	RR	1.06 (0.91–1.23)	0.4548	0	0.41–2.76	0.2323	*P* > 0.05	NA	V
Ou et al. (40)	Viruses	CMV-a	AD	6	680/356	OR	1.17 (0.67–2.05)	0.5869	35.5	0.29–4.66	0.0035	*P* < 0.05	NA	V
Ou et al. (40)		CMV-b control studies	AD	2	1569/453	RR	1.46 (0.67–3.15)	0.3377	84.4	NA	NA	*P* < 0.05	NA	V
Ou et al. (40)		HHV-6	AD	4	456/204	OR	3.97 (2.04–7.75)	5.14E-05	0	0.92–17.22	0.9463	*P* < 0.05	NA	IV
Ou et al. (40)		VZV	AD	3	143/70	OR	1.12 (0.45–2.78)	0.8075	0	0.00–410.31	0.4734	*P* > 0.05	NA	V
Ou et al. (40)		EBV	AD	2	297/112	OR	1.45 (1.01–2.09)	0.0468	0	NA	NA	*P* < 0.05	NA	IV
Wu et al. (13)		HSV-1	AD	21	3566/1338	OR	1.41 (1.13–1.75)	0.0021	1.7	1.07–1.85	0.2229	*P* > 0.05	NA	IV
Tooran et al. (41)	Parasites	Toxoplasmosis	AD	8	3239/360	OR	1.53 (1.07–2.19)	0.0191	19.1	0.76–3.08	0.0261	*P* < 0.05	NA	IV
Ou et al. (40)	Chlamydia	Cpn	AD	11	740/389	OR	4.39 (1.78–10.81)	0.0013	71.4	0.30–64.21	0.2644	*P* < 0.05	Yes/no	IV
Maheshwari et al. (42)	Other microorganisms	Spirochetes-a	AD	13	1204/723	OR	10.65 (3.40–33.42)	5.00E-05	51.6	0.41–279.54	0.2348	*P* < 0.05	Yes/no	IV
Maheshwari et al. (42)		Spirochetes-b	AD	9	460/236	OR	4.46 (2.33–8.55)	6.46E-06	0	2.04–9.77	0.4491	*P* < 0.05	NA	IV
Ou et al. (40)		Herpesviridae family-a	AD	34	2050/1083	OR	1.41 (1.14–1.74)	0.0014	14.4	0.85–2.33	0.6695	*P* < 0.05	No/no	IV
Ou et al. (40)		Herpesviridae family-b	AD	9	12166/1406	RR	1.13 (0.91–1.40)	0.2827	56.8	0.61–2.09	0.7641	*P* > 0.05	NA	V
Leira et al. (43)		Periodontal Disease	AD	3	822/204	RR	1.79 (1.15–2.78)	0.01	25	0.04–86.14	0.6202	NA	NA	IV
Nadim et al. (44)	Other microorganisms	Periodontal Disease	Dementia	11	227098/21298	RR	1.65 (1.28–2.13)	0.0001	97	0.52–5.22	0.6219	*P* < 0.05	yes/no	III
Muzambi et al. (45)		Sepsis(remove studies with high risk of bias)	Dementia	3	503938/50624	HR	1.60 (1.19–2.16)	0.0019	95.6	0.04–69.99	0.7505	*P* < 0.05	yes/no	IV
Shindler-Itskovitch et al. (46)	Bacteria	HP	Dementia	7	85715/18145	OR	1.71 (1.17–2.48)	0.0053	76.1	0.53–5.46	0.3315	*P* < 0.05	yes/no	IV

CI, confidence interval; PI, prediction intervals; HP, Helicobacter pylori; PD, Parkinson's disease; OR, odds ratio; HCV, hepatitis C virus; NA, not available; HBV, hepatitis B virus; HSV, herpes virus; AD, Alzheimer's disease; RR, risk ratio; CMV, cytomegalovirus; HHV-6, Human herpes virus type 6; VZV, varicella zoster virus; EBV, Epstein Barr virus; HSV-1, herpes simplex virus type 1; HR, Hazard ratio; Toxoplasmosis-a, Toxoplasmosis(IgG antibodies); Toxoplasmosis-b, Toxoplasmosis(IgM antibodies); Cpn, Chlamydophila pneumonia; SIBO, Small intestinal bacterial overgrowth; HP-a, HP in case control studies; HP-b, HP in cohort studies; CMV-a, CMV in case control studies; CMV-b, CMV in cohort and nested case control studies; Spirochetes-a, all studies; Spirochetes-b, conservative studies; Herpesviridae family-a, Herpesviridae family in case control studies; Herpesviridae family-b, Herpesviridae family in cohort and nested case control studies.

#### Associations for PD

A total of 19 of the 37 meta-analyses examined associations for PD. Among them, 3 meta-analyses (16%) were rated as level II evidence (highly evidence); these relationships were HP, infection, and bacteria, respectively. Additionally, 6 meta-analyses (32%) were evaluated as level IV evidence, including malassezia, HCV, *Chlamydophila pneumoniae*, Chicken POX, small intestinal bacterial overgrowth (SIBO), and viruses, among which Chicken POX was a protective factor for PD but with a weak evidence level. The remaining 10 meta-analyses (53%) demonstrated no significant evidence, including measles, HBV, German measles, HSV, infuenza, mumps, scarlet fever, whooping cough, toxoplasmosis-IgG, and toxoplasmosis-IgM.

#### Associations for AD

A total of 15 of the 37 meta-analyses examined associations for AD. Specifically, 9 of 15 meta-analyses (60%) were evaluated as level IV evidence (weak evidence). These risk factors included Herpesviridae family infection (in case-control studies), Chlamydia pneumoniae, HHV-6, and EBV, Spirochetes, conservative Spirochetes, periodontal disease, toxoplasmosis, and HSV-1. The remaining meta-analyses, 6 of 15 associations (40%), were rated as having no evidence, involving the risk factors of the Herpesviridae family (in cohort and nested case-control studies), CMV (in case-control studies), CMV (in cohort and nested case-control studies), VZV, HP (in case-control studies), and HP (in cohort studies).

#### Associations for all types of dementia

Three of the 37 meta-analyses examined associations for all types of dementia. Periodontal disease was rated as level III evidence (suggestive evidence). The other two risk factors are sepsis and HP, which are rated as level IV evidence.

## Discussions

An umbrella review was conducted to provide a comprehensive overview of the currently available meta-analyses of microbiological factors and neurodegenerative disorders. The evidence for microbial factors associated with the incidence of neurodegenerative diseases was summarized, and the evidence level was evaluated.

None of the 37 independent associations in the 19 meta-analyses and systematic reviews identified were rated high-quality evidence. Among the single microbial risk factors, only HP was considered highly suggestive evidence (class II) related to the development of PD. Infection and bacteria were also considered highly suggestive evidence (class II) for PD. However, the two risk factors were synthesized by many microbial risk factors instead of single microbial risk factors. Since this comprehensive conclusion was significantly affected by different research scopes, the reliability of such evidence was very low and needed careful interpretation. Only one piece of evidence was assessed as the level of suggestion evidence (class III): the periodontal disease was the risk factor for all types of dementia. Meanwhile, other microbiological factors were assessed as weak (class IV).

Significant heterogeneity existed in more than half of the studies. Six studies were influenced by small-study effects. Considering that heterogeneity reflects real differences between included studies, findings should be carefully interpreted when small studies have significant effects or when heterogeneity is large. P curves in only 10 studies exhibited a yes or no type, while other studies have the possibility of p-hacking existence.

### Comparison with other studies and possible explanations

In our umbrella analysis, HP infection is the highly suggestive evidence level of PD pathogenesis, which is the highest level of evidence in our investigation of neurodegenerative diseases. Moreover, the other three meta-analyses in the literature were included in this paper. Although they did not become an item in the evidence table after the CAA method, the conclusions of these three meta-analyses are consistent with our evidence (39, 47, 48). Besides, a considerable number of clinical studies suggest that HP not only has a higher infection rate in PD patients than in the control group (49–52) but also leads to an aggravation of PD symptoms, especially the fluctuation of motor symptoms (23, 49, 53–56). The mean UPDRS-III score in patients with PD was significantly reduced after HP eradication therapy (31, 47, 53, 57). Animal studies have revealed that the pathogenesis of PD may start from the gastrointestinal tract. The mouse model of rotenone poisoning can simulate the pathogenesis of PD; gastrointestinal symptoms in these mouse models precede the onset of motor symptoms and are pathologically consistent (4). In other words, α-synuclein pathology in the colon appears before the aggregation of a-Syn in the midbrain (24), consistent with the notion that gastrointestinal symptoms in PD patients are prodromal symptoms of eventual motor dysfunction (58). Furthermore, the eventual onset of motor symptoms is likely to be achieved through the bidirectional action of the micobiome-gut-brain axis (4, 59, 60). Therefore, the pathogenesis of the disease may be multifactorial. Meanwhile, a synergy between these factors ultimately leads to neuronal destruction in genetically susceptible individuals with PD (61). Braak's theory implies that the disease originates in the gut and subsequently spreads to the brain through the vagus nerve (28). This notion was confirmed by injecting α-synuclein into the gut of healthy rats, which eventually induced lesions in the vagus nerve and brainstem (62, 63). Patients with full truncal vagotomy are at a lower risk of developing PD, confirming the existence of the vagal route from the side (25, 29, 64). Similarly, McGee et al. proposed the hypothesis that HP may produce a toxin affecting the gut microbiota (58). Several toxins produced by HP may induce PD. Altschuler speculated that HP may be synthesizing a substance similar to MPTP (65). Wunder suggested that the glycosylation of host cholesterol by HP may be toxic (66). Another pathway is through the activation of the immune system and the release of pro-inflammatory factors (67). HP can persist in the gut and produce a chronic inflammatory state inducing the secretion of many of its mediators, comprising pro-inflammatory cytokines (tumor necrosis factor- α, interleukin-1β, interleukin-6, and interferon γ), which were elevated in PD (30, 68). These pro-inflammatory molecules can enter the brain through the gut-brain axis by means of leakage from the blood-brain barrier (69, 70). Ultimately, these proinflammatory cytokines and neurotoxic agents can enter the brain and stimulate microglia, triggering neuroinflammatory responses and promoting the progression of PD (58). Regarding treatment, a recently published article summarized a series of anti-infective drugs exerting neuroprotective effects through multiple mechanisms, such as interfering with synuclein aggregation, inhibiting neuroinflammation, reducing oxidative stress, and preventing cell death. The gut and microbes may become a new entry point for the prevention and treatment of PD (71). Our study reveals that HP eradication therapy is reasonable in patients with PD. Moreover, HP eradication therapy is recommended for high-risk groups of PD because the clinical process of diagnosing and eradicating HP is easy and inexpensive.

Our umbrella review demonstrated that the evidence level of microbial risk factors associated with AD was weak, and no evidence of high grade was discovered. Single microbial risk factors included HSV-1, HHV-6, EBV, spirochete, Toxoplasma gondii, and chlamydia pneumoniae. Among them, the risk factors with more than 1,000 people included in the study were HSV-1, spirochete, and Toxoplasma gondii.

Four studies within our search scope have performed meta-analyses on whether HSV-1 infection is a risk factor for the development of AD. After the data were processed by the CAA method, the most recent meta-analysis with the largest number of original documents was selected as the basis for the evaluation of the level of evidence. However, the conclusions of the other three meta-analyses were not entirely consistent with our selection. For example, the meta-analysis by Steel et al. indicated that HSV-1 infection increased the risk of AD, while the meta-analysis by Warren-Gash et al. drew the opposite conclusion (11, 12). A meta-analysis by Ou et al. grouped the association between HSV-1 infection and the risk of AD. Particularly, 18 combined case-control studies showed that HSV-1 infection increased the risk of AD, whereas 4 prospective cohort studies and 1 nested case-control study came to the opposite conclusion when combined (40). The reasons for the inconsistent conclusions are described as follows. First, case-control studies are susceptible to confounding factors while failing to determine the temporal relationship between HSV-1 infection and AD, which can easily lead to false-positive results. Second, HSV-1 carriers may not increase the risk of AD, and HSV-1 reactivation may increase the risk of AD. Animal studies have shown that AD-like pathology can be observed after mice are repeatedly infected with HSV-1 from the viral reactivation cycle, and cognitive deficits are increased and irreversible after 7 reactivation cycles (72). Moreover, HSV-1-IgM antibody positivity is a marker of viral reactivation and is associated with the risk of AD (26, 35, 73). Third, it may be related to whether the APOE-ε4 gene is carried. Itzhaki et al. detected the brain tissue and APOE-ε4 gene of AD and non-AD patients, respectively; revealing that the combination of the HSV-1 gene and the APOE-ε4 allele in the brain is a strong risk factor for AD, and neither of these features alone increases the risk of AD (74). In conclusion, the relationship between HSV-1 infection and AD risk is uncertain, and a large prospective cohort study should be performed to further determine the relationship.

Including risk estimates from all studies or excluding conservative risk estimates with extreme OR values all suggest that Spirochetes infection is associated with AD risk. Over the past few decades, Miklossy et al. have extensively researched the relationship between spirochetes and AD and made a systematic presentation at the International Association of Gerontology and Geriatrics (IAGG) Congress held in July 2017 in San Francisco (6). Herrera-Landero et al. found that patients with positive IgG antibodies to *Borrelia burgdorferi* were at increased risk of developing AD (75). Moreover, Miklossy et al. tested all types of spirochetes in the brains of 83 AD patients and 31 normal people, and analyzed 680 brain and blood samples. In AD, more than 91.1% (451/495) of samples were positive, while a surprising 185 control samples were negative (6, 76). Mechanistically, syphilitic dementia was first discovered to reproduce the filamentous pathological features of AD (77). Several types of spirochetes have been intensively studied since then. Herbert B. Allen believed that the spirochetes form biofilms, which stimulate the innate immune system to produce toll-like receptor 2, contributing to the formation of NF-kB and TNF-a to kill the spirochetes in the biofilm. Nevertheless, the biofilm cannot be penetrated. NF-kB promotes the production of Aß. Although Aß is antimicrobial, it cannot penetrate biofilms, and its accumulation leads to the disruption of nerve cells in the brain and reproduces the pathology of AD (78). A recent study published by Senejani et al. discovered that Borrelia-positive aggregates co-localized with amyloid and phosphorylated tau protein markers in brain tissue of AD patients (79). Based on the above mechanisms, researchers have proposed the hypothesis of using penicillin (PCN) and biofilm-dispersed drugs to prevent and treat AD. However, its effectiveness should be further verified (80). Some researchers disagree with this. A 30-year cohort study from Denmark did not reveal an increased long-term risk of dementia in patients with Lyme disease (81), which may be related to the young age of the patients enrolled and the failure to follow them up throughout life. In our evaluation of the evidence level, the *P*-value can reach moderate evidence. Unfortunately, it is still evaluated as weak evidence since the number of cases is small, and the level of evidence may increase as the number of cases increases. According to our research results and the explanation of the mechanism by many researchers, we believe that spirochetes are related to the pathogenesis of AD. In the future, the pathogenesis and prevention strategies of AD need to be deeply investigated.

Our umbrella review identified Toxoplasma as a weakly graded risk factor for AD. Bayani et al. also performed a meta-analysis of the relationship between Toxoplasma infection and AD and observed a slightly significant association, consistent with our results (37). A case-control study by Kusbeci et al. suggested that IgG antibodies to *Toxoplasma gondii* were 44.1 and 24.3% in AD patients and healthy controls, respectively. The difference in serum antibodies was statistically significant, and a positive correlation between toxoplasmosis and AD was considered (82). Mahmoudvand et al. reported that BALB/c mice developed AD-like symptoms after Toxoplasma infection, and the learning and memory function of the mice was impaired (83). Additionally, Torres et al. demonstrated that Toxoplasma infection induced two major features of AD in the brains of C57BL/6 male and female mice (Aβ immunoreactivity and Tau protein hyperphosphorylation), and infected mice exhibited marked neuronal death (84). However, Toxoplasma infection is not associated with AD, as indicated in many studies (33, 85, 86). There are even studies suggesting that Toxoplasma infection has a protective effect on AD (87). The reasons for different conclusions are provided as follows. (1) Whether there is a susceptibility gene for AD remains unclear. Yahya et al. reported that Toxoplasma-positive patients are at higher risk of developing dementia in the presence of APOE-ε4 (88), while most studies did not consider genetic susceptibility factors. (2) The species of Toxoplasma gondii are different. Cabral et al. revealed that infection with Toxoplasma gondii type II had better protection compared with strains of Toxoplasma gondii types I and III (89). (3) Many studies only rely on serological antibody tests, and it may be difficult to find the relationship between nervous system infections and AD because of the lack of research on brain tissue samples. (4) Some studies involve a small number of cases, and the findings may not be comprehensive enough. In conclusion, the relationship between Toxoplasma gondii and AD is currently controversial. Thus, more scientific and larger studies should be designed to examine the relationship between Toxoplasma infection and AD.

Our umbrella analysis demonstrated that Periodontal Disease is a risk factor for dementia with a moderate level of evidence. An epidemiological survey from Japan implied that poor oral health was significantly associated with cognitive impairment (90). AD is the most common cause of dementia. A study from Sweden pointed out a strong association between periodontitis and both early cognitive impairment and AD (91). Cohort studies that can present a temporal relationship are more suggestive of a causal relationship, regardless of numerous cross-sectional studies linking periodontitis with dementia. A large 11-year cohort study of 182,747 patients with periodontitis by Lee et al. indicated that subjects with more severe or untreated periodontitis are at greater risk of dementia (92). Similarly, Demmer et al. conducted a large multicenter (*n* = 8,275) cohort study. Their findings also revealed that periodontal disease was associated with dementia events (34). Additionally, a recent meta-analysis by Guo et al. disclosed that there is a correlation between periodontitis and cognitive impairment, and moderate or severe periodontitis is a risk factor for dementia, consistent with our findings (93). The exact molecular mechanism of the involvement of periodontitis in the pathogenicity of dementia remains ambiguous, and the possible explanations are detailed as follows. First, periodontitis (gum disease) is a persistent low-grade inflammation caused by pathogenic microorganisms that results in the release of inflammatory factors (c-reactive protein, tumor necrosis factor, interleukin-1, interleukin-6, α-1antichymotrypsin), and inflammatory factors can enter the blood-brain barrier and affect the initiation or activation of microglia in the brain due to the pathogenesis of dementia (94). Second, the microorganisms causing periodontitis and their by-products can exert toxic effects on neurons in the brain. Dominy et al. uncovered the presence of *Pseudomonas gingivalis* DNA and antigens of gingivalin in the brains of AD patients and elaborated that they play a central role in the pathogenesis of AD (95, 96). Even Dominy et al. affirmed that gingivalin inhibitors could block amyloidosis triggered by oral infection of *Bacillus gingivalis* in mice (96). This provided a new direction for the treatment of dementia. Meanwhile, a double-blind, placebo-controlled phase II/III study of a bacterial protease inhibitor against *Porphyromonas gingivalis* in periodontal disease is underway for the treatment of mild to moderate AD (97). Although dementia may be multifactorial, interventions targeting periodontitis are warranted given the epidemiological evidence and our findings.

### Strengths and limitations

The main strength of the present umbrella review is the comprehensive overview of the published meta-analyses on the association between microbiological factors and the risk of neurodegenerative disorders. To our knowledge, we are the first to evaluate the methodological quality of the meta-analyses and the level of evidence for all these associations. The AMSTAR2 instrument was employed to assess the methodological quality of the included meta-analyses. Additionally, the CAA method was adopted to quantify the overlap of meta-analysis. The highest-quality and most recent meta-analysis was selected under the combination of these two methods, avoiding double counting and selection bias. Moreover, all meta-analyses were recalculated using the random-effects model, and the level of evidence was evaluated for each meta-analysis. Consequently, a comprehensive, up-to-date evidence hierarchy was provided for microbiological risk factors of neurodegenerative diseases, contributing to reliable clinical guidance and potential research directions.

Some limitations exist in our umbrella review. First, all the meta-analyses included were based on observational studies. However, confounding factors were inevitable as the data in our study were derived from observational studies (98). Second, Gray literature and systematic reviews without meta-analyses were not considered in this study, leading to some bias. Third, there are some original studies on microbial risk factors and neurodegenerative diseases that may have been published between the search deadline and the publication of the results of this study. Some biases would be induced because these results were not considered. Fourth, the World Health Organization (WHO) claims that Coronavirus disease 2019 (COVID-19) has become a global pandemic on 11 March 2020 (99). Moreover, many researchers have conducted a meta-analysis on the relationship between COVID-19 and neurodegenerative diseases. However, due to the short appearance time of COVID-19, the main research topic is the adverse outcomes of patients with neurodegenerative diseases infected with COVID-19, rather than the etiological relationship (47, 48, 100). Therefore, this paper did not treat COVID-19 as a risk factor for neurodegenerative diseases. Fifth, some indicators, such as Egger's test *p*-value, 95% PI, and evidential value for P-curve cannot be calculated due to the small number of original studies included in some meta-analyses. Therefore, the evidence level of this part could not be evaluated.

## Conclusions

A comprehensive overview of current meta-analyses of microbial risk factors and neurodegenerative diseases is presented in this paper. Although numerous studies suggest that multiple microbes are associated with neurodegenerative diseases, the overall level of evidence is not high. It is revealed that HP infection is a risk factor and Class II evidence for PD, and periodontitis is a risk factor and Class III evidence for dementia, laying a foundation for HP removal and periodontitis treatment and enlightening a new direction for research on the treatment of PD and dementia. More high-quality research is required in the future.

## Data availability statement

The original contributions presented in the study are included in the article/Supplementary material, further inquiries can be directed to the corresponding authors.

## Author contributions

JL conceived the study. JL, CX, XW, and DJ designed the study. XW and TL collected data and performed the analysis with input from XZ, RW, SG, FY, YW, and QT. XW and JL wrote the manuscript with contributions from all authors. All authors critically revised and approved the manuscript.

## Conflict of interest

The authors declare that the research was conducted in the absence of any commercial or financial relationships that could be construed as a potential conflict of interest.

## Publisher's note

All claims expressed in this article are solely those of the authors and do not necessarily represent those of their affiliated organizations, or those of the publisher, the editors and the reviewers. Any product that may be evaluated in this article, or claim that may be made by its manufacturer, is not guaranteed or endorsed by the publisher.
