# Supplementary material for: Association between microbiological risk factors and neurodegenerative disorders: An umbrella review of systematic reviews and meta-analyses
Source: Front Psychiatry. 2022 Sep 23;13:991085. doi: 10.3389/fpsyt.2022.991085 (PMC9537612; doi:10.3389/fpsyt.2022.991085)
Supplement: Supplementary file 1 [file Data_Sheet_1.PDF]

APPENDICES

Appendix 1: PRISMA checklist

Appendix 2: Search strategy for Pubmed

Appendix 3: List of excluded studies and reasons for their exclusion

Appendix 4: AMSTAR 2 quality appraisal scores

Appendix 5: Citation matrices for meta-analyses with overlapping associations

Appendix 6: Overlapping associations

Appendix 7: List of included studies

Appendix 1: PRISMA checklist

Section/topic		Checklist item
Title	Item 1	Identify the report as a systematic review
Abstract	Item 2	See the PRISMA 2020 for Abstracts checklist
Rationale	Item 3	Describe the rationale for the review in the context of existing knowledge
Objectives	Item 4	Provide an explicit statement of the objective(s) or question(s) the review addresses
Eligibility criteria	Item 5	Specify the inclusion and exclusion criteria for the review and how studies were grouped for the syntheses
Information sources	Item 6	Specify all databases, registers, websites, organisations, reference lists, and other sources searched or consulted to identify studies. Specify the date when each source was last searched or consulted
Search strategy	Item 7	Present the full search strategies for all databases, registers, and websites, including any filters and limits used
Selection process	Item 8	Specify the methods used to decide whether a study met the inclusion criteria of the review, including how many reviewers screened each record and each report retrieved, whether they worked independently, and, if applicable, details of automation tools used in the process
Data collection process	Item 9	Specify the methods used to collect data from reports, including how many reviewers collected data from each report, whether they worked independently, any processes for obtaining or confirming data from study investigators, and, if applicable, details of automation tools used in the process
Data items	Item 10a	List and define all outcomes for which data were sought. Specify whether all results that were compatible with each outcome domain in each study were sought (for example, for all measures, time points, analyses), and, if not, the methods used to decide which results to collect
	Item 10b	List and define all other variables for which data were sought (such as participant and intervention characteristics, funding sources). Describe any assumptions made about any missing or unclear information
Study risk of bias assessment	Item 11	Specify the methods used to assess risk of bias in the included studies, including details of the tool(s) used, how many reviewers assessed each study and whether they worked independently, and, if applicable, details of automation tools used in the process
Effect measures	Item 12	Specify for each outcome the effect measure(s) (such as risk ratio, mean difference) used in the synthesis or presentation of results
Synthesis methods	Item 13a	Describe the processes used to decide which studies were eligible for each synthesis (such as tabulating the study intervention characteristics and comparing against the planned groups for each synthesis (item #5))
	Item 13b	Describe any methods required to prepare the data for presentation or synthesis, such as handling of missing summary statistics or data conversions
	Item 13c	Describe any methods used to tabulate or visually display results of individual studies and syntheses
	Item 13d	Describe any methods used to synthesise results and provide a rationale for the choice(s). If meta-analysis was performed, describe the model(s), method(s) to identify the presence and extent of statistical heterogeneity, and software package(s) used
	Item 13e	Describe any methods used to explore possible causes of heterogeneity among study results (such as subgroup analysis, meta regression)
	Item 13f	Describe any sensitivity analyses conducted to assess robustness of the synthesised results
Reporting bias assessment	Item 14	Describe any methods used to assess risk of bias due to missing results in a synthesis (arising from reporting biases)
Certainty assessment	Item 15	Describe any methods used to assess certainty (or confidence) in the body of evidence for an outcome
Study selection	Item 16a	Describe the results of the search and selection process, from the number of records identified in the search to the number of studies included in the review, ideally using a flow diagram
	Item 16b	Cite studies that might appear to meet the inclusion criteria, but which were excluded, and explain why they were excluded
Study characteristics	Item 17	Cite each included study and present its characteristics
Risk of bias in studies	Item 18	Present assessments of risk of bias for each included stud
Results of individual studies	Item 19	For all outcomes, present for each study (a) summary statistics for each group (where appropriate) and (b) an effect estimate and its precision (such as confidence/credible interval), ideally using structured tables or plots
Results of syntheses	Item	For each synthesis, briefly summarise the characteristics and risk of bias among contributing studies

	20a	
	Item 20b	Present results of all statistical syntheses conducted. If meta-analysis was done, present for each the summary estimate and its precision (such as confidence/credible interval) and measures of statistical heterogeneity. If comparing groups, describe the direction of the effect
	Item 20c	Present results of all investigations of possible causes of heterogeneity among study results
	Item 20d	Present results of all sensitivity analyses conducted to assess the robustness of the synthesised results
Risk of reporting biases in syntheses	Item 21	Present assessments of risk of bias due to missing results (arising from reporting biases) for each synthesis assessed
Certainty of evidence	Item 22	Present assessments of certainty (or confidence) in the body of evidence for each outcome assessed
Discussion	Item 23a	Provide a general interpretation of the results in the context of other evidence
	Item 23b	Discuss any limitations of the evidence included in the review
	Item 23c	Discuss any limitations of the review processes used
	Item 23d	Discuss implications of the results for practice, policy, and future research
Registration and protocol	Item 24a	Provide registration information for the review, including register name and registration number, or state that the review was not registered
	Item 24b	Indicate where the review protocol can be accessed, or state that a protocol was not prepared
	Item 24c	Describe and explain any amendments to information provided at registration or in the protocol
Support	Item 25	Describe sources of financial or non financial support for the review, and the role of the funders or sponsors in the review
Competing interests	Item 26	Declare any competing interests of review authors
Availability of data, code, and other materials	Item 27	Report which of the following are publicly available and where they can be found: template data collection forms; data extracted from included studies; data used for all analyses; analytic code; any other materials used in the review

Appendix 2: Search strategy for Pubmed

#1	"Cochrane Database Syst Rev"[Journal] OR "Systematic review"[Title/Abstract] OR "Systematic reviews"[Title/Abstract] OR meta analysis[Title/Abstract] OR meta analyses[Title/Abstract] OR metaanalysis[Title/Abstract] OR metaanalyses[Title/Abstract] OR metanalysis[Title/Abstract] OR met analysis[Title/Abstract] OR metanalyses[Title/Abstract] OR met analyses[Title/Abstract] OR data pooling[Title/Abstract] OR data poolings[Title/Abstract] OR clinical trial overview[Title/Abstract] OR clinical trial overviews[Title/Abstract] OR clinical trial overviews[Title/Abstract] OR meta-analysis[Title/Abstract] OR meta-analyses[Title/Abstract] OR "Systematic review"[Publication Type] OR "Systematic Reviews as Topic"[MeSH Terms] OR Meta-Analysis as Topic[MeSH Terms] OR Meta-Analysis[Publication Type]
#2	Neurodegenerative Diseases[MeSH Terms] OR Neurodegenerative Diseases[Title/Abstract] OR Alzheimer Disease[MeSH Terms] OR Alzheimer Disease[Title/Abstract] OR Parkinson Disease[MeSH Terms] OR Parkinson Disease[Title/Abstract] OR Multiple System Atrophy[MeSH Terms] OR Multiple System Atrophy[Title/Abstract] OR Lewy Body Disease[MeSH Terms] OR Lewy Body Disease[Title/Abstract] OR Dementia[MeSH Terms] OR Dementia[Title/Abstract] OR Motor Neuron Disease[MeSH Terms] OR Motor Neuron Disease[Title/Abstract]
#3	Infections[MeSH Terms] OR Organisms Category[MeSH Terms] OR gingivitis[Title/Abstract] OR Gum infection[Title/Abstract] OR Gingipains[Title/Abstract] OR Porphyromonas gingivalis[Title/Abstract] OR PGINGIVALIS[Title/Abstract] OR Herpes virus[Title/Abstract] OR Chlamydia[Title/Abstract] OR Chlamydophila[Title/Abstract] OR Pneumonia[Title/Abstract] OR pneumoniae[Title/Abstract] OR EB virus[Title/Abstract] OR Epstein-Barr virus[Title/Abstract] OR spirochete[Title/Abstract] OR treponemata[Title/Abstract] OR spirochaete[Title/Abstract] OR fungus[Title/Abstract] OR Herpes simplex virus 1[Title/Abstract] OR intestinal flora[Title/Abstract] OR H pylori[Title/Abstract] OR helicobacter pylori[Title/Abstract] OR Virus[Title/Abstract] OR toxoplasma gondii[Title/Abstract] OR microorganism[Title/Abstract] OR Bacterial Infections[Title/Abstract] OR Gingivitis[Title/Abstract]
#4	#1 AND #2 AND #3

Appendix 3: List of excluded studies and reasons for their exclusion

	1st Author	Year	Title	Reason for Exclusion
1	Priscila Cunha Nascimento	2019	Association Between Periodontitis and Cognitive Impairment in Adults:A Systematic Review	No quantitative synthesis
2	Nathan D. Nuzum	2020	Gut microbiota differences between healthy older adults and individuals with Parkinson's disease: A systematic review	
3	Andrea Ticinesi	2018	Gut microbiota, cognitive frailty and dementia in older individuals: a systematic review	
4	Jeffrey M. Boertien	2019	Increasing Comparability and Utility of Gut Microbiome Studies in Parkinson's Disease:A Systematic Review	
5	Cátia Almeida, PharmD	2020	Influence of gut microbiota dysbiosis on brain function: a systematic review	
6	Pallavi P. Tonsekar	2017	Periodontal disease, tooth loss and dementia: Is there a link? A systematic review	
7	Rosalind M. Tucker	2020	Role of Helicobacters in Neuropsychiatric Disease: A Systematic Review in Idiopathic Parkinsonism	
8	Mario Dioguardi	2020	The Role of Periodontitis and Periodontal Bacteria in the Onset and Progression of Alzheimer's Disease: A Systematic Review	
9	Bei Wu, PhD	2016	Association Between Oral Health and Cognitive Status: A Systematic Review	
10	Matthew R. Nangle	2019	Oral Health and Cognitive Function in Older Adults: A Systematic Review	
11	Michelle L. Wright, RN, PhD	2018	Potential Role of the Gut Microbiome in ALS: A Systematic Review	
12	Michael Doulberis	2020	Alzheimer's disease and gastrointestinal microbiota; impact of Helicobacter pylori infection involvement	Reviews
13	Francis Mawanda	2013	Can Infections Cause Alzheimer's Disease?	
14	Sara Gerhardt	2018	Changes of Colonic Bacterial Composition in Parkinson's Disease and Other Neurodegenerative Diseases	
15	Rita Khoury	2020	Deciphering Alzheimer's disease: predicting new therapeutic strategies via improved understanding of biology and pathogenesis	
16	Gagandeep Kaur	2020	Dysregulation of the Gut-Brain Axis, Dysbiosis and Influence of numerous factors on Gut Microbiota associated Parkinson's Disease	
17	Mohamed Elfil, MD	2020	Implications of the Gut Microbiome in Parkinson's Disease	
18	Vanessa de J. R. De-Paula	2018	Relevance of gut microbiota in cognition, behaviour and Alzheimer's disease	
19	Wen Gao, RN, MSN	2020	The Gut Microbiome as a Component of the Gut–Brain Axis in Cognitive Health	
20	Chun-Hung Chang	2020	d-glutamate and Gut Microbiota in Alzheimer's Disease	
21	B. Daly	2017	Evidence summary: the relationship between oral health and dementia	
22	P. BOLLERO	2017	ORAL HEALTH AND IMPLANT THERAPY IN PARKINSON'S PATIENTS: REVIEW	
23	Suzanne Delwel	2017	Oral hygiene and oral health in older people with dementia:a comprehensive review with focus on oral soft tissues	
24	Learn-Han Lee	2018	DISSECTING THE GUT AND BRAIN:POTENTIAL LINKS BETWEEN GUT MICROBIOTA IN DEVELOPMENT OF ALZHEIMER'S DISEASE?	The profile
25	Y. Wang	2017	Gut microbiota are related to Parkinson's disease	
26	Analia Luiza Porto Viana	2019	HERPES SIMPLEX VIRUS AND ALZHEIMER'S DISEASE: THE PRESENT STATE OF EVIDENCE	
27	Roy W Jones	2001	Inflammation and Alzheimer's disease	
28	AyuniYussof	2020	A meta- analysis of the effect of binge drinking on the oral microbiome and its relation to Alzheimer's disease	Incorrect exposure

**29**	Steven Bradburn	2019	Neuroinflammation in mild cognitive impairment and Alzheimer’s disease: A meta-analysis	
**30**	Chang-Kai Chen	2017	Association between chronic periodontitis and the risk of Alzheimer’s disease: a retrospective, population-based, matchedcohort study	Original research
**31**	Hiroshi Nishiwaki, MD	2020	Meta-Analysis of Gut Dysbiosis in Parkinson’s Disease	Part of the country

Appendix 4: AMSTAR 2 quality appraisal scores

Item No	1	2*	3	4*	5	6	7*	8	9*	10	11*	12	13*	14	15*	16	Overall Rating
Karn Wijarnpreecha 2017	No	No	Yes	Partial Yes	Yes	Yes	No	Yes	Yes	Yes	Yes	Yes	Yes	Yes	Yes	Yes	moderate
Hui Wang2020	No	No	Yes	Partial Yes	Yes	No	No	Yes	Yes	Yes	Yes	Yes	Yes	Yes	Yes	Yes	moderate
Lei Meng2019	No	No	Yes	No	Yes	Yes	No	Yes	No	Yes	Yes	No	Yes	Yes	Yes	Yes	critically low
Ya-Nan Ou2020	No	No	Yes	Partial Yes	Yes	Yes	No	Yes	Yes	Yes	Yes	Yes	Yes	Yes	Yes	Yes	moderate
Priya Maheshwari 2016	No	Partial Yes	Yes	Partial Yes	No	No	No	Yes	No	No	Yes	Yes	Yes	Yes	No	No	low
Rutendo Muzambi2020	Yes	Yes	Yes	Partial Yes	Yes	Yes	No	Yes	Yes	No	Yes	Yes	Yes	Yes	Yes	No	moderate
Nayeri Chegeni Tooran2019	No	Yes	Yes	Partial Yes	Yes	Yes	No	Yes	Yes	No	Yes	Yes	Yes	Yes	Yes	No	moderate
Masomeh Bayani2019	No	Yes	Yes	Partial Yes	Yes	Yes	No	Yes	Yes	Yes	Yes	Yes	Yes	Yes	Yes	Yes	moderate
Zonglei Zhou2019	No	No	Yes	Partial Yes	Yes	Yes	No	Yes	Yes	Yes	Yes	Yes	Yes	Yes	Yes	Yes	moderate
Rizwan Nadim2020	Yes	No	Yes	Partial Yes	Yes	Yes	No	Yes	Yes	Yes	Yes	Yes	Yes	Yes	Yes	Yes	moderate
Yago Leira2017	No	Yes	Yes	Partial Yes	Yes	Yes	No	Yes	Yes	Yes	Yes	Yes	Yes	Yes	No	Yes	moderate
David Jonathan R. Gusman2018	Yes	Yes	Yes	Partial Yes	Yes	Yes	No	Yes	Yes	Yes	Yes	Yes	Yes	Yes	No	Yes	moderate
Ariah J. Steel2015	No	Yes	Yes	Partial Yes	No	No	No	Yes	No	No	Yes	Yes	No	Yes	Yes	No	low
CharlotteWarren-Gash 2019	No	Yes	Yes	Partial Yes	Yes	Yes	No	Yes	Yes	No	Yes	Yes	Yes	Yes	Yes	No	moderate
Donghong Wu2020	No	Yes	Yes	Partial Yes	Yes	Yes	No	Yes	Yes	Yes	Yes	Yes	Yes	Yes	Yes	Yes	moderate
Efthimios Dardiotis2018	No	No	Yes	No	Yes	Yes	No	Yes	No	Yes	Yes	Yes	Yes	Yes	Yes	Yes	critically low
Xiaoli	No	No	Yes	Partial	Yes	Yes	No	Yes	No	Yes	Yes	Yes	Yes	Yes	Yes	Yes	low

Shen2017				Yes													
Pengfei Fu2019	No	No	Yes	No	No	No	No	Yes	Yes	Yes	Yes	Yes	Yes	Yes	Yes	Yes	low
Tali Shindler-Itskovitch2016	No	No	Yes	Partial Yes	Yes	Yes	No	Yes	No	Yes	Yes	Yes	Yes	Yes	Yes	Yes	low

*: critical domains. Item 1: Did the research questions and inclusion criteria for the review include the components of PICO? Item 2: Did the report of the review contain an explicit statement that the review methods were established prior to the conduct of the review and did the report justify any significantdeviations from the protocol? Item 3: Did the review authors explain their selection of the study designs for inclusion in the review? Item 4: Did the review authors use a comprehensive literature search strategy? Item 5: Did the review authors perform study selection in duplicate? Item 6:Did the review authors perform data extraction in duplicate? Item 7: Did the review authors provide a list of excluded studies and justify the exclusions? Item 8: Did the review authors describe the included studies in adequate detail? Item 9: Did the review authors use a satisfactory technique for assessing the risk of bias (RoB) in individual studies that were included in the review? Item 10: Did the review authors report on the sources of funding for the studies included in the review? Item 11: If meta-analysis was performed, did the review authors use appropriate methods for statistical combination of results? Item 12: If meta-analysis was performed, did the review authors assess the potential impact of RoB in individual studies on the results of the meta-analysis or other evidence synthesis? Item 13: Did the review authors account for RoB in primary studies when interpreting/discussing the results of the review? Item 14: Did the review authors provide a satisfactory explanation for, and discussion of, any heterogeneity observed in the results of the review? Item 15: If they performed quantitative synthesis did the review authors carry out an adequate investigation of publication bias (small study bias) and discuss its likely impact on the results of the review? Item 16: Did the review authors report any potential sources of conflict of interest, including any funding they received for conducting the review?

Appendix 5: Citation matrices for meta-analyses with overlapping associations

A.HCV and PD

Systematic reviews: HCV(exposure)	Karn Wijarnpreecha 2017	Hui Wang2020
**Overlapping association**	**PD**	**PD**
**Primary Study**		
Wu2015	X	X
Tasi2015	X	X
Kim2016	X	X
Golabi2017	X	X
Pakpoor2017	X	X
Lilach2019		X
Su2019		X
Total (No of publications per review)	5	7
Grand Total (N)	12	
Rows (r)	7	
Columns (c)	2	
Corrected covered area (CCA)	71%	

HCV = hepatitis C virus, PD = Parkinson's disease

Formula for calculating the corrected covered area, CCA (%) = N-r / rc-r: Where N = number of included publications (sum of checked boxes), r = number of rows (primary publications), c = number of columns (number of reviews).

B.HP and PD

Systematic reviews: HP(exposure)	Hui Wang2020	Efthimios Dardiotis2018	Xiaoli Shen2017	Pengfei Fu2019
**Overlapping association**	**PD**	**PD**	**PD**	**PD**
**Primary Study**				
Charlett1999	X	X	X	X
Dobbs2000	X	X	X	X
Charlett2009	X	X	X	X
Nieisen2012	X	X	X	X
Blaecher2013	X	X	X	X
Nafisah2013	X	X	X	X
Bu2015	X	X	X	X
Tsolaki2015	X	X	X	X
Huang2017	X			X
Fasano2013		X		
Ethymiou2017		X		
Total (No of publications per review)	9	10	8	9
Grand Total(N)	36			
Rows (r)	11			
Columns (c)	4			
Corrected covered area (CCA)	76%			

HP = Helicobacter pylori, PD = Parkinson's disease

Formula for calculating the corrected covered area, CCA (%) = N-r / rc-r: Where N = number of included publications (sum of checked boxes), r = number of rows (primary publications), c = number of columns (number of reviews).

C.HSV-1 and AD

Systematic reviews: HSV1(exposure)	Ya-Nan Ou2020	Ya-Nan Ou2020	Ariah J. Steel2015	CharlotteWarren-Gash 2019	Donghong Wu2020
**Overlapping association**	**AD**	**AD**	**AD**	**AD**	**AD**
**Primary Study**					
Bu2014	X				X
Mancuso2013	X		X		X
Kobayashi2012	X		X		X
Wozniak2004	X		X		X
Mori2004	X		X	X	X
Hemling2003	X		X	X	X
Marques2001	X		X		X
Beffert1998-21	X		X	X	X
Lin1998	X		X		
Beffert1998-28	X		X		X
Itabashi1997	X		X	X	X
Lin1997	X		X		X
Itzhaki1997	X		X		X
Lin1996	X		X	X	X
Bertrand1993	X		X	X	
Jamieson1992	X		X	X	X
Ounanian1990	X		X		X
Mann1983	X		X		X
Torniainen2018		X			
Lovheim2015		X			
Lovheim2014		X			
Barnes2014		X			
Letenneur2008		X	X		X
Cheon2001				X	
Deatly1990				X	
Jamieson1991				X	
Kittur1992				X	
Lin1994				X	
Lin2002a				X	
Roberts1986				X	
Tayior1986				X	
Wozniak2009				X	
Agostini2018					X
Linard2019					X
Lovheim2017					X
Mancuso-b2016					X
Total (No of publications per review)	18	5	18	16	21
Grand Total(N)	78				

Rows (r)	36				
Columns (c)	5				
Corrected covered area (CCA)	29%				

HSV-1 = herpes simplex virus type 1, AD = Alzheimer's disease

Formula for calculating the corrected covered area, CCA (%) = N-r / rc-r: Where N = number of included publications (sum of checked boxes), r = number of rows (primary publications), c = number of columns (number of reviews).

D.Herpesviridae family and AD

Systematic reviews:	Ya-Nan Ou2020	Ya-Nan Ou2020	Ariah J. Steel2015
**Overlapping association**	**AD**	**AD**	**AD**
**Primary Study**			
Bu2014	X		
Mancuso2013	X		
Kobayashi2012	X		X
Wozniak2004	X		X
Mori2004	X		X
Hemling2003	X		X
Marques2001	X		X
Beffert1998	X		X
Lin1998	X		X
Beffert1998	X		X
Itabashi1997	X		X
Lin1997	X		X
Itzhaki1997	X		X
Lin1996	X		X
Bertrand1993	X		X
Jamieson1992	X		X
Ounanian1990	X		X
Mann1983	X		X
Bu2014	X		
Mancuso2013	X		X
Westman2013	X		X
Larain2013	X		X
Lin2002	X		X
Ounanian1990	X		X
Carbone2013	X		X
Ounanian1990	X		X
Carbone2013	X		X
Wozniak2004	X		X
Hemling2003	X		X
Lin2002	X		X
Hemling2003	X		X
Lin1997	X		
Ounanian1990	X		X
Lin2002	X		X
Torniainen2018		X	
Lovheim2015		X	
Lovheim2014		X	
Barnes2014		X	

Letenneur2008		X	X
Lovheim2018		X	
Barnes2014		X	
Carbone2013		X	X
Carbone2013		X	X
Total (No of publications per review)	34	9	33
Grand Total(N)	76		
Rows (r)	43		
Columns (c)	3		
Corrected covered area (CCA)	38%		

AD = Alzheimer's disease

Formula for calculating the corrected covered area, CCA (%) = N-r / rc-r: Where N = number of included publications (sum of checked boxes), r = number of rows (primary publications), c = number of columns (number of reviews).

E.Chlamydophila pneumoniae and AD

Systematic reviews: CPN(exposure)	Ya-Nan Ou2020	Priya Maheshwari2016
**Overlapping association**	**AD**	**AD**
**Primary Study**		
Bu2014	X	
Hammond2010	X	X
Ecemis2010	X	X
Paradowski2007	X	X
Gerard2006	X	X
Yamamoto2005	X	X
Wozniak2003	X	X
Taylor2002	X	X
Ring2000	X	X
Nochlin1999	X	X
Balin1998	X	X
Mahony		X
Total (No of publications per review)	11	11
Grand Total(N)	22	
Rows (r)	12	
Columns (c)	2	
Corrected covered area (CCA)	83%	

AD = Alzheimer's disease

Formula for calculating the corrected covered area, CCA (%) = N-r / rc-r: Where N = number of included publications (sum of checked boxes), r = number of rows (primary publications), c = number of columns (number of reviews).

F.CMV and AD

Systematic reviews: CMV(exposure)	Ya-Nan Ou2020	Ya-Nan Ou2020	Ariah J. Steel2015
**Overlapping association**	**AD**	**AD**	**AD**
**Primary Study**			
Bu2014	X		
Mancuso2013	X		
Westman2013	X		X
Larain2013	X		X
Lin2002	X		X
Ounanian1990	X		X
Lovheim2018		X	
Barnes2014		X	
Total (No of publications per review)	6	2	4
Grand Total(N)	12		
Rows (r)	8		
Columns (c)	3		
Corrected covered area (CCA)	25%		

CMV = cytomegalovirus, AD = Alzheimer's disease

Formula for calculating the corrected covered area, CCA (%) = N-r / rc-r: Where N = number of included publications (sum of checked boxes), r = number of rows (primary publications), c = number of columns (number of reviews).

G.HHV-6 and AD

Systematic reviews: HHV-6(exposure)	Ya-Nan Ou2020	Ariah J. Steel2015
**Overlapping association**	**AD**	**AD**
**Primary Study**		
Carbone2013b	X	X
Carbone2013c		X
Wozniak2004	X	X
Hemling2003	X	X
Lin2002	X	X
Total (No of publications per review)	4	5
Grand Total(N)	9	
Rows (r)	5	
Columns (c)	2	
Corrected covered area (CCA)	80%	

HHV-6 = Human herpes virus type 6, AD = Alzheimer's disease

Formula for calculating the corrected covered area, CCA (%) = N-r / rc-r: Where N = number of included publications (sum of checked boxes), r = number of rows (primary publications), c = number of columns (number of reviews).

H.VZV and AD

Systematic reviews: VZV(exposure)	Ya-Nan Ou2020	Ariah J. Steel2015
**Overlapping association**	**AD**	**AD**
**Primary Study**		
Hemling2003	X	X
Lin1997	X	
Ounanian1990	X	X
Total (No of publications per review)	3	2
Grand Total(N)	5	
Rows (r)	3	
Columns (c)	2	
Corrected covered area (CCA)	67%	

VZV = varicella zoster virus, AD = Alzheimer's disease

Formula for calculating the corrected covered area, CCA (%) = N-r / rc-r: Where N = number of included publications (sum of checked boxes), r = number of rows (primary publications), c = number of columns (number of reviews).

I.EBV and AD

Systematic reviews: EBV(exposure)	Ya-Nan Ou2020	Ariah J. Steel2015
**Overlapping association**	**AD**	**AD**
**Primary Study**		
Carbone2013	X	X
Ounanian1990	X	X
Total (No of publications per review)	2	2
Grand Total(N)	4	
Rows (r)	2	
Columns (c)	2	
Corrected covered area (CCA)	100%	

EBV = Epstein Barr virus, AD = Alzheimer's disease

Formula for calculating the corrected covered area, CCA (%) = N-r / rc-r: Where N = number of included publications (sum of checked boxes), r = number of rows (primary publications), c = number of columns (number of reviews).

J.HP and AD

Systematic reviews: HP(exposure)	Ya-Nan Ou2020	Ya-Nan Ou2020	Pengfei Fu2019
**Overlapping association**	**AD**	**AD**	**AD**
**Primary Study**			
Bu2014	X		
Shiota2011	X		X
Kounlouras2006	X		X
Nagga2003	X		
Beydoun2018		X	
Fani2018		X	X
Huang2014		X	X
Tsolaki2015			X
Total (No of publications per review)	4	3	5
Grand Total(N)	12		
Rows (r)	8		
Columns (c)	3		
Corrected covered area (CCA)	25%		

HP = Helicobacter pylori, AD = Alzheimer's disease

Formula for calculating the corrected covered area, CCA (%) = N-r / rc-r: Where N = number of included publications (sum of checked boxes), r = number of rows (primary publications), c = number of columns (number of reviews).

K.Spirochetes and AD

Systematic reviews: Spirochetes (exposure)	Ya-Nan Ou2020	Priya Maheshwari2016	Priya Maheshwari2016
**Overlapping association**	**AD**	**AD**	**AD**
**Primary Study**			
Riviere2002	X	X	X
McLaughlin1999	X	X	X
Miklossy1993	X	X	
Miklossy1994		X	
MacDonald1987		X	X
Pappolla1989		X	X
MacDonald2006		X	X
Marques2000		X	X
Galbussera2008		X	X
Marquard2012		X	X
Pappolla1989a		X	X
Miklossy1995		X	
Miklossy1998		X	
Total (No of publications per review)	3	13	9
Grand Total(N)	25		
Rows (r)	13		
Columns (c)	3		
Corrected covered area (CCA)	46%		

AD = Alzheimer's disease

Formula for calculating the corrected covered area, CCA (%) = N-r / rc-r: Where N = number of included publications (sum of checked boxes), r = number of rows (primary publications), c = number of columns (number of reviews).

L.Sepsis and Dementia

Systematic reviews: Sepsis (exposure)	Rutendo Muzambi2020	Rutendo Muzambi2020
**Overlapping association**	**dementia**	**dementia**
**Primary Study**		
Guerra2012	X	X
Mawanda2016	X	X
Shah2013	X	
Chou2017		X
Total (No of publications per review)	3	3
Grand Total(N)	6	
Rows (r)	4	
Columns (c)	2	
Corrected covered area (CCA)	50%	

Formula for calculating the corrected covered area, CCA (%) = N-r / rc-r: Where N = number of included publications (sum of checked boxes), r = number of rows (primary publications), c = number of columns (number of reviews).

M.Toxoplasmosis and AD

Systematic reviews: Toxoplasmosis (exposure)	Nayeri Chegeni Tooran2019	Masomeh Bayani2019
**Overlapping association**	**AD**	**AD**
**Primary Study**		
Alvarado2006	X	
Kusbeci2011	X	X
Cong1 2015	X	
Cong2 2015	X	
Menati Rashno2016	X	X
Mahami2016	X	X
Zaki2016	X	
Perry2016	X	X
Flegr and Horaceka2017	X	
Total (No of publications per review)	9	4
Grand Total(N)	13	
Rows (r)	9	
Columns (c)	2	
Corrected covered area (CCA)	44%	

AD = Alzheimer's disease

Formula for calculating the corrected covered area, CCA (%) = N-r / rc-r: Where N = number of included publications (sum of checked boxes), r = number of rows (primary publications), c = number of columns (number of reviews).

N.Toxoplasmosis（latent infection IgG antibodies) and PD

Systematic reviews: Toxoplasmosis (exposure)	Masomeh Bayani2019	Zonglei Zhou2019
**Overlapping association**	**PD**	**PD**
**Primary Study**		
Fallahi2017	X	X
Alvarado2017	X	X
Celik2010	X	X
Celik2013	X	X
El Gendy2017	X	
Mahami2016	X	X
Miman2010	X	X
Ramezani2016	X	X
Total (No of publications per review	8	7
Grand Total(N)	15	
Rows (r)	8	
Columns (c)	2	
Corrected covered area (CCA)	88%	

PD = Parkinson's disease

Formula for calculating the corrected covered area, CCA (%) = N-r / rc-r: Where N = number of included publications (sum of checked boxes), r = number of rows (primary publications), c = number of columns (number of reviews).

O.Toxoplasmosis （acute infection IgM antibodies) and PD

Systematic reviews: Toxoplasmosis (exposure)	Masomeh Bayani2019	Zonglei Zhou2019
**Overlapping association**	**PD**	**PD**
**Primary Study**		
Fallahi2017	X	X
Alvarado2017	X	X
El Gendy2017	X	
Ramezani2016 A		X
Total (No of publications per review	3	3
Grand Total(N)	6	
Rows (r)	4	
Columns (c)	2	
Corrected covered area (CCA)	50%	

PD = Parkinson's disease

Formula for calculating the corrected covered area, CCA (%) = N-r / rc-r: Where N = number of included publications (sum of checked boxes), r = number of rows (primary publications), c = number of columns (number of reviews).

P.Periodontal disease and Dementia

Systematic reviews: periodontal disease (exposure)	Rizwan Nadim2020	Rizwan Nadim2020	David Jonathan R. Gusman2018
**Overlapping association**	**dementia**	**dementia**	**dementia**
**Primary Study**			
Arrive2012 moderate PD in low education	X	X	
Arrive2012 severe PD in low education	X	X	
Arrive2012 moderate PD in high education	X	X	
Arrive2012 severe PD in high education	X	X	
Tzeng2016	X	X	
Chen2017	X	X	
Lee YL2017 mild PD with intensive treatment	X	X	
Lee YL2017 moderate PD with tooth extraction	X	X	
Lee YL2017 severe PD without treatment	X	X	
Lee YL2017	X	X	
Gil-Montoya2015 moderate PD	X	X	X
Gil-Montoya2015 severe PD	X	X	X
Holmer2018	X	X	
Chu2014 moderate PD		X	
Chu2014 severe PD		X	
De Souza2014 mild PD		X	
De Souza2014 moderate PD		X	
De Souza2014 severe PD		X	
Bramanti2015		X	
Jureti2016 moderate PD		X	
Jureti2016 severe PD		X	
Cestari2016			X
Martande2014			X
Rai2012			X
Total (No of publications per review	13	21	5
Grand Total(N)	39		
Rows (r)	24		
Columns (c)	3		
Corrected covered area (CCA)	31%		

Formula for calculating the corrected covered area, CCA (%) = N-r / rc-r: Where N = number of included publications (sum of checked boxes), r = number of rows (primary publications), c = number of columns (number of reviews).

Appendix 6: Overlapping associations

Index of overlapping associations	Study ID	AMSTAR rating	exposure	Outcome	Corrected covered area (CCA)	Decision to retain ✓= Yes ✗= No
1	Karn Wijarnpreecha 2017<br>Hui Wang2020	moderate<br>moderate	HCV	PD	71%very high	✗<br>✓
2	Hui Wang2020<br>Efthimios Dardiotis2018<br>Xiaoli Shen2017<br>Pengfei Fu2019	moderate<br>critically low<br>low<br>low	HP	PD	76%very high	✓<br>✗<br>✗<br>✗
3	Ya-Nan Ou2020<br>Ya-Nan Ou2020<br>Ariah J. Steel2015<br>CharlotteWarren-Gash 2019<br>Donghong Wu2020	moderate<br>moderate<br>low<br>moderate<br>moderate	HSV-1	AD	29%very high	✗<br>✗<br>✗<br>✗<br>✓
4	Ya-Nan Ou2020<br>Ya-Nan Ou2020<br>Ariah J. Steel2015	moderate<br>moderate<br>low	Herpesviridae family	AD	38%very high	✓<br>✓<br>✗
5	Ya-Nan Ou2020<br>Priya Maheshwari2016	moderate<br>low	Chlamydophila pneumoniae	AD	83%very high	✓<br>✗
6	Ya-Nan Ou2020<br>Ya-Nan Ou2020<br>Ariah J. Steel2015	moderate<br>moderate<br>low	CMV	AD	25%very high	✓<br>✓<br>✗
7	Ya-Nan Ou2020<br>Ariah J. Steel2015	moderate<br>low	HHV-6	AD	100%very high	✓<br>✗
8	Ya-Nan Ou2020<br>Ariah J. Steel2015	moderate<br>low	VZV	AD	67%very high	✓<br>✗
9	Ya-Nan Ou2020<br>Ariah J. Steel2015	moderate<br>low	EBV	AD	100%very high	✓<br>✗
10	Ya-Nan Ou2020<br>Ya-Nan Ou2020<br>Pengfei Fu2019	moderate<br>moderate<br>low	HP	AD	25%very high	✓<br>✓<br>✗
11	Ya-Nan Ou2020<br>Priya Maheshwari2016<br>Priya Maheshwari2016	moderate<br>low<br>low	Spirochetes	AD	46%very high	✗<br>✓<br>✓
12	Rutendo Muzambi2020<br>Rutendo Muzambi2020	moderate<br>moderate	Sepsis	dementia	50%very high	✗<br>✓
13	Nayeri Chegeni Tooran2019<br>Masomeh Bayani2019	moderate<br>moderate	Toxoplasmosis	AD	44%very high	✓<br>✗

**14**	Masomeh Bayani2019<br><br>Zonglei Zhou2019	moderate<br><br>moderate	Toxoplasmosis （latent infection IgG antibodies)	PD	88%very high	✓<br><br>✗
**15**	Masomeh Bayani2019<br><br>Zonglei Zhou2019	moderate<br><br>moderate	Toxoplasmosis （acute infection IgM antibodies)	PD	50%very high	✓<br><br>✗
**16**	Rizwan Nadim2020<br><br>Rizwan Nadim2020<br><br>David Jonathan R. Gusman2018	moderate<br><br>moderate<br><br>moderate	periodontal disease	dementia	31%very high	✗<br>✓<br>✗

HCV hepatitis C virus, PD Parkinson's disease, HP Helicobacter pylori, HSV-1 herpes simplex virus type 1, AD Alzheimer's disease, CMV cytomegalovirus, HHV-6 Human herpes virus type 6, VZV varicella zoster virus, EBV Epstein Barr virus

Appendix 7: List of included studies

	1st Author	Year	Title
1	Karn Wijarnpreecha	2017	Hepatitis C virus infection and risk of Parkinson’s disease: a systematic review and meta-analysis
2	Hui Wang	2020	Bacterial, viral, and fungal infection-related risk of Parkinson's disease: Meta-analysis of cohort and case–control studies
3	Lei Meng	2018	Impact of infection on risk of Parkinson’s disease: a quantitative assessment of case-control and cohort studies
4	Ya-Nan Ou	2020	Associations of Infectious Agents with Alzheimer’s Disease: A Systematic Review and Meta-Analysis
5	Priya Maheshwari	2016	Bacterial Infection Increases the Risk of Alzheimer’s Disease: An Evidence-Based Assessment
6	Rutendo Muzambi	2020	Common Bacterial Infections and Risk of Dementia or Cognitive Decline: A Systematic Review
7	Nayeri Chegeni Tooran	2019	Is Toxoplasma gondii a potential risk factor for Alzheimer's disease? A systematic review and meta-analysis
8	Zonglei Zhou	2019	The Association between Toxoplasma gondii Infection and Risk of Parkinson’s Disease: A Systematic Review and Meta-Analysis
9	Masomeh Bayani	2019	Toxoplasma gondii infection and risk of Parkinson and Alzheimer diseases: a systematic review and meta-analysis on observational studies
10	Rizwan Nadim	2020	Infuence of periodontal disease on risk of dementia: a systematic literature review and a meta- analysis
11	Yago Leira	2017	Is Periodontal Disease Associated with Alzheimer’s Disease? A Systematic Review with Meta-Analysis
12	Ariah J. Steel	2015	Herpes Viruses Increase the Risk of Alzheimer’s Disease: A Meta-Analysis
13	CharlotteWarren-Gash	2019	Human herpesvirus infections and dementia or mild cognitive impairment: a systematic review and meta-analysis
14	Donghong Wu	2020	The association between herpes simplex virus type 1 infection and Alzheimer’s disease
15	Efthimios Dardiotis	2018	H. pylori and Parkinson’s disease: meta-analyses including clinical severity
16	Xiaoli Shen	2017	Association of Helicobacter pylori infection with Parkinson’s diseases: A meta-analysis
17	Pengfei FU	2019	Association of intestinal disorders with Parkinson’s disease and Alzheimer’s disease: A systematic review and meta-analysis
18	Tali Shindler-Itskovitch	2016	A Systematic Review and Meta-Analysis of the Association between Helicobacter pylori Infection and Dementia
19	David Jonathan R. Gusman	2018	Periodontal disease severity in subjects with dementia: a systematic review and meta-analysis
